# High-performance triboelectric nanogenerators based on Ag-doped ZnO loaded electrospun PVDF nanofiber mats for energy harvesting and healthcare monitoring

**DOI:** 10.1038/s41598-025-87148-8

**Published:** 2025-01-27

**Authors:** Hema Malini Venkatesan, Anand Prabu Arun

**Affiliations:** Department of Chemistry, School of Advanced Sciences, Vellore Institute of Technology, Vellore, 632014 Tamil Nadu India

**Keywords:** Silver doped zinc oxide, Poly(vinylidene fluoride), Electrospinning, Triboelectric nanogenerator, Energy harvesting, Healthcare monitoring, Energy science and technology, Materials science

## Abstract

This study investigates the potential of zinc oxide (ZnO) and Ag-doped zinc oxide (Ag-ZnO) nanoparticles (NPs) (1, 3 and 5 wt%) electrospun into poly(vinylidene fluoride) (PVDF) based triboelectric nanogenerators (TENGs) to harness electrical energy from ambient mechanical vibrations. ZnO and Ag-ZnO NPs were developed using a co-precipitation method. 3 wt% Ag-ZnO doping was optimized to exhibit a higher *β*-crystalline phase in PVDF (PAZ3). The electrospun ZnO and Ag-ZnO loaded PVDF nanofiber mats (NFMs) served as tribo-negative layer, while thermoplastic polyurethane (TPU) served as tribo-positive layer in the TENG device fabrication. The electrical measurement results revealed that pristine PVDF/TPU-based TENG device exhibited lower performance (*V*_oc_ = 9.0 V and *I*_sc_ = 0.6 µA) compared to the optimized PAZ3/TPU-based TENG (*V*_oc_ = 51 V and *I*_sc_ = 1.2 µA), and utilized for concurrent applications such as powering over 10 light-emitting diodes, energy harvesting and healthcare monitoring applications. Overall, the study not only reveals the significant potential of PAZ3/TPU-based TENG for excellent output performance, but also provides a new insight into next-generation clean, affordable and sustainable energy harvesting materials.

**Supplementary Information:**

The online version contains supplementary material available at 10.1038/s41598-025-87148-8.

## Introduction

The United Nations has established seventeen sustainable development goals (SDGs) to accelerate global efforts towards an eco-friendly and zero-waste future. The seventh among SDGs focuses on affordable renewable energy, aiming to guarantee universal access to reliable, modern, and affordable sustainable energy sources^1^. Our proposed research directly supports this goal by accomplishing effective, economical, and renewable mechanical energy harvesting solutions. Various methods have been reported on converting sustainable renewable and ambient energy into electrical energy^2,3^. Mechanical energy harvesters, such as electromagnetic^4^ and piezoelectric generators^5^ can produce high output. However, they have not performed efficiently with low-scale vibrations due to the low magnitude of mechanical stimuli. In this context, triboelectric nanogenerators (TENGs) have attracted attention for their capability to efficiently convert low-frequency vibrations into electricity from the waste surrounding mechanical energy^6,7^. TENGs operate based on two mechanisms: contact electrification and electrostatic induction^8^. Due to their versatile and simple designs, wide range of material options, and low fabrication costs, TENGs have become increasingly popular in the field of energy harvesting and self-sustainable sensors^9,10^.

Wang et al. were the first to report a TENG energy harvesting device in 2012, commonly referred to as the Wang generator^11^. Since then, numerous researchers have explored various combinations of triboelectric materials to capture mechanical energy. Zou et al.^12^ performed an extensive analysis of tribo-materials, emphasizing the importance of selecting ideal pairings according to the triboelectric series, as shown in the supplementary material Fig. S1. To attain enhanced electrical performance in TENGs, it is crucial to select materials with significant differences in electron affinity and maximize the frictional contact area of the materials. For instance, TENGs demonstrate superior electrical performance when the highest triboelectric positive (TP) layer material is paired with the lowest triboelectric negative (TN) layer material^13^.

Materials like poly(vinylidene fluoride) (PVDF) nanofiber mats (NFMs) have emerged as a suitable option for a TN material. PVDF is highly versatile because it can generate electrical energy when bent or when it comes into contact with other material owing to the triboelectric phenomenon^14^. The polar *β*-phase present in PVDF allows it to produce a strong electrical signal under external force. Additionally, PVDF has a high affinity for attracting electrons, which helps create a powerful electric field and retain an electrical charge for an extended period. Due to these advantageous properties, PVDF is frequently used as the TN frictional layer in devices like TENGs^15^. The efficacy of TENGs primarily relies on the overall charges present on the surface of the triboelectric material. To enhance TENG performance, it is crucial to improve the charges over the material surfaces^16^. Methods such as chemical treatments, surface patterning, incorporating electron-capturing materials, and corona charging have been employed for this purpose. However, these techniques can be complex, expensive, and their effects may not be long-lasting^2^. Compared to fiber extrusion, spin-coating, melt-casting, and phase inversion methods, electrospinning stands out as an easy and cost-effective method to produce aligned and uneven surface in PVDF NFMs, which in turn, increases the effective contact area between two triboelectric layers, thereby enhancing the output performance of TENGs^2,17^. Consequently, fabricating PVDF nanocomposites NFMs *via* electrospinning presents an optimistic approach to fabricate highly efficient TENGs^18^.

Moreover, incorporating nanoparticles (NPs) into pristine PVDF is a promising method to boost the efficiency of NFMs by enhancing the *β*-phase. The high surface-to-volume ratio of NPs promotes greater interaction at the polymer-filler interface, thereby increasing the *β*-phase fraction of the polymer matrix. These filler materials induce more *β*-phase in the PVDF nanocomposites, substantially improving their ability to generate electricity from contact and movement. Therefore, the incorporation of NPs fillers with PVDF significantly enhances its triboelectric properties^2^. Due to ease of synthesis, compatibility, and multi-functionality, fillers including metal oxide NPs, semiconductors, and 2D materials has been extensively studied to enhance the triboelectric output efficiency. Nanofillers like BaTiO_3_^19^, MoS_2_^20^, TiO_2_^16^, CuO^21^, and ZnO^5^ have been employed as effective nucleating and charge trapping agents in TENGs. These fillers trap charges at the interface, thereby increasing the amount of transferred charge per unit area in PVDF-based nanocomposites^22^. Among the aforementioned materials, ZnO is an II-VI n-type semiconductor, which has gained considerable attention due to its multifunctional properties and wide range of applications in electronics, sensors, and actuators, particularly for enhanced sensing performance^23^. A key difference between ZnO and Ag-doped ZnO lies in in their conductivity and dielectric properties. Though ZnO exhibits good dielectric properties, its electric conductivity is relatively low. Doping ZnO with Ag enhances its conductivity and dielectric properties owing to its ability to create regions of strong electric fields and improve its electrical characteristics. The addition of Ag into ZnO introduces defects and excess free charge carriers within the nanostructure, which synergistically enhances charge accumulation over the triboelectric frictional layer. As a highly conductive metal, Ag increases the overall surface charges on electrospun Ag-ZnO/PVDF NFs, which promotes faster charge transfer between the TP and TN frictional layer, thereby minimizing the triboelectric loss, which is crucial for enhance the overall electric performance of the TENG device^24,25^. Further, ZnO and Ag-ZnO could be easily prepared by the facile co-precipitation method^26^. A detailed comparison of previously reported literature is given in the supplementary material Table S1. Recently, Zhu et al.^5^ have reported a CNF-ZnO/PVDF-based piezoelectric nanogenerator (PENG) designed for energy harvesting and environmental monitoring applications. However, the usage of higher filler loading yielded lesser output voltage (*V*_oc_ = 11.8 V). To the best of our knowledge, no prior literature has explored a flexible Ag-ZnO doped electrospun PVDF NFMs based TN frictional layer, stacked with TPU as the TP layer based TENG, which is adding the novelty aspect to this work.

In this study, we investigated and refined the impact of changing concentrations of ZnO and Ag-ZnO (ranging from 0 to 5 wt%) on the *β*-phase nucleation and its electrical properties. The optimal doping concentration was found to be 3 wt% of Ag-ZnO for PVDF (PAZ3), and its results were compared with ZnO doped PVDF-based TENG devices. Finally, the optimized PAZ3 based TN layer is combined with TPU based TP layer for fabricating a TENG device in the contact-separation (CS) mode. The output efficiency of the PAZ3/TPU TENG device was analyzed. The optimized TENG device was designed to demonstrate the realistic feasibility of mechanical energy harvesting, energizing low-power electronics such as LEDs, and HCM applications. The results are discussed in detail in the following sections.

## Experimental section

### Materials

This study utilized an analytical-grade metal acetate and metal nitrate precursor salts, such as zinc(II) acetate dihydrate (Zn(CH_3_COO)_2_.2H_2_O, *M*_w_ = 219 g/mol, $\:\ge\:$99% purity) and silver(II) nitrate (AgNO_3_, *M*_w_ = 170 g/mol, $\:\ge\:$99% purity). Additionally, sodium hydroxide (NaOH, *M*_w_ = 40 g/mol, $\:\ge\:$98% purity), ethanol (C_2_H_6_O, *M*_w_ = 46 g/mol, $\:\ge\:$99.5% purity) and distilled water (DW) were used for the NPs synthesis. All the chemicals were procured from Sigma-Aldrich, USA. The following chemicals were obtained from South Korea; PVDF powder (CH_2_CF_2_)_n_, Kynar^®^761, *M*_w_ = 370,000 g/mol), *N*,* N*-dimethylformamide (DMF) (C_3_H_7_NO, *M*_w_ = 73 g/mol, $\:\ge\:$99.8% purity), and acetone (C_3_H_6_O, *M*_w_ = 58 g/mol, $\:\ge\:$99.5% purity) from Atochem, Ltd. The one-side adhesive polyester fabric electrode coated with nickel-copper (Ni-Cu) electrode was purchased from Solueta Co. Ltd. Melt-blown TPU was purchased from J.S. Inc. All the chemicals were used in their state, without any further purification.

## Synthesis of ZnO NPs

**Fig. 1:**
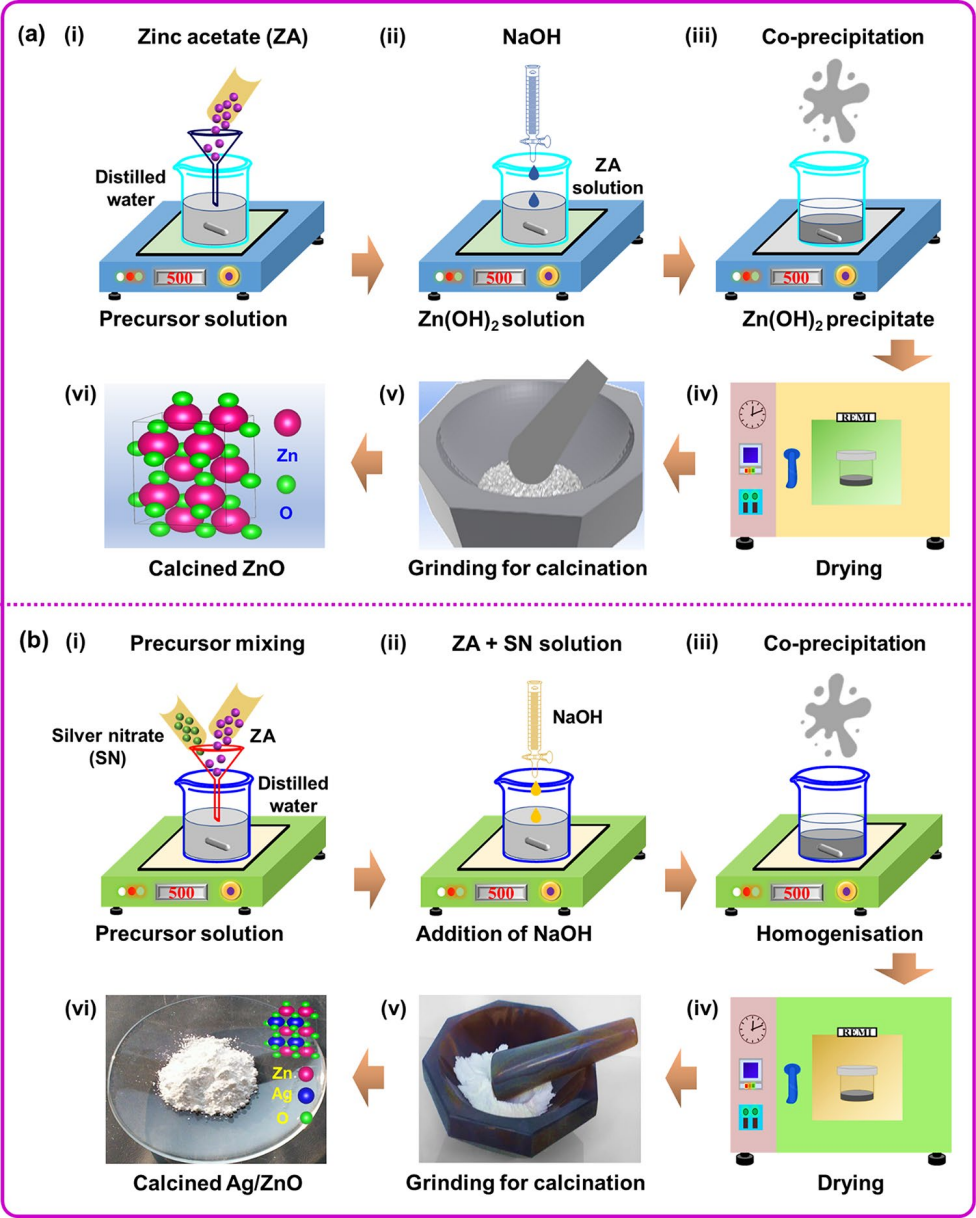
(**a** and **b**) A schematic step-by-step synthesis process representation of ZnO and Ag-ZnO NPs using the facile co-precipitation method.

In two separate beakers, 1 M zinc acetate dihydrate (ZAD) and 2 M sodium hydroxide (NaOH) was each dissolved in 50 mL of distilled water (DW). The resulted ZAD precursor solution and precipitating agent solution (NaOH) were stirred at a constant 500 rpm for 30 min. Subsequently, the NaOH solution was added dropwise to the ZAD solution, with the mixture being stirred at constant 500 rpm for 4 h. The white precipitate was left untouched throughout the night to ensure complete homogenization and precipitation. The resulting precipitate was collected by centrifuging at 4000 rpm and then washed multiple times with DW. To reduce aggregation and eliminate trapped water molecule, the sample was further washed with ethanol. The sample was then dried in hot air oven at approximately 100 ˚C overnight. Lastly, the sample was annealed at 500 ˚C for 2 h to form crystalline ZnO NPs. The schematic illustration of ZnO NPs synthesis were shown in Fig. 1a. The chemical reaction for the synthesis of ZnO at room temperature is given as follows^27^.

Step 1: Precursor and precipitating agent dissolution in DW

$$\begin{aligned} & \left( {\text{i}} \right)\:{\text{Zn}}({\text{CH}}_{3} {\text{COO}})_{2} 0.2{\text{H}}_{2} {\text{O}}\:({\text{s}})\: \to \:{\text{Zn}}^{{2 + }} \:({\text{aq}}) + 2{\text{CH}}_{3} {\text{COO}}^{ - } \:({\text{aq}}) + \:2{\text{H}}_{2} {\text{O}}\:({\text{l}}) \\ & \left( {{\text{ii}}} \right)\:2{\text{NaOH}}\:\left( {\text{s}} \right)\: \to \:2{\text{Na}}^{ + } \:\left( {{\text{aq}}} \right) + 2{\text{OH}}^{ - } \:\left( {{\text{aq}}} \right)\: \\ \end{aligned}$$

Step 2: Adding NaOH to the precursor solution

$$\:\left[2{\text{N}\text{a}}^{+}\:\left(\text{a}\text{q}\right)+2{\text{O}\text{H}}^{-}\:\left(\text{a}\text{q}\right)\right]\:+\left[{\text{Z}\text{n}}^{2+}\:\left(\text{a}\text{q}\right)+{2{\text{C}\text{H}}_{3}\text{C}\text{O}\text{O}}^{-}\:\left(\text{a}\text{q}\right)+2{\text{H}}_{2}\text{O}\:\left(\text{l}\right)\right]\to\:{\text{Z}\text{n}\left(\text{O}\text{H}\right)}_{2}\:\left(\text{s}\right)+2{\text{C}\text{H}}_{3}\text{C}\text{O}\text{O}\text{N}\text{a}\:\left(\text{a}\text{q}\right)+2{\text{H}}_{2}\text{O}\:\left(\text{l}\right)$$

Step 3: Calcination

$$\:{\text{Z}\text{n}\left(\text{O}\text{H}\right)}_{2}\:\left(\text{s}\right)\begin{array}{c}500\text{℃}\\\:\to\:\\\:2\:\text{h}\end{array}\:\text{Z}\text{n}\text{O}\:\left(\text{s}\right)+\:{\text{H}}_{2}\text{O}\:(\uparrow\:)$$

## Synthesis of Ag-ZnO NPs

The Ag-doping was carried out by following the same procedure as described in ZnO NPs synthesis. Along with the 1 M ZAD precursor, 1 M of silver nitrate (SN) was added. These two precursor solutions were mixed thoroughly under a constant stirring of 500 rpm for 30 min. Subsequently, the 1 M NaOH solution was added dropwise to the ZAD and SN solution mixture, with the mixture being stirred at room temperature at a constant 500 rpm for 4 h. The obtained white precipitate was allowed to settle down overnight to ensure complete the homogenization and precipitation. The resulting white precipitate was collected by centrifugation at 4000 rpm and washed multiple times with DW and ethanol. The obtained sample was then oven-dried at 100 ˚C for overnight. Finally, the sample was annealed at 500 ˚C for 2 h to form crystalline Ag-ZnO NPs. The schematic illustration of ZnO NPs synthesis were shown in Fig. 1b. The chemical reaction for the synthesis of ZnO and Ag-ZnO (at room temperature) is given as follows^27^.

Step 1: Precursor and precipitating agent dissolution in DW

$$\begin{aligned} & \left( {\text{i}} \right)\:{\text{Zn}}({\text{CH}}_{3} {\text{COO}})_{2} 0.2{\text{H}}_{2} {\text{O}}\:({\text{s}})\: \to \:{\text{Zn}}^{{2 + }} \:({\text{aq}}) + 2{\text{CH}}_{3} {\text{COO}}^{ - } \:({\text{aq}}) + \:2{\text{H}}_{2} {\text{O}}\:({\text{l}}) \\ & \left( {{\text{ii}}} \right)\:{\text{AgNO}}_{3} \:\left( {\text{s}} \right)\: \to \:{\text{Ag}}^{ + } \:\left( {{\text{aq}}} \right) + {\text{NO}}_{3} ^{ - } \:\left( {{\text{aq}}} \right)\: \\ & \left( {{\text{iii}}} \right)\:{\text{NaOH}}\:\left( {\text{s}} \right)\: \to \:{\text{Na}}^{ + } \:\left( {{\text{aq}}} \right) + {\text{OH}}^{ - } \:\left( {{\text{aq}}} \right) \\ \end{aligned}$$

Step 2: Adding NaOH to the precursor solution.

(i) Reaction between ZAD and NaOH

$$\:\left[2{\text{N}\text{a}}^{+}\:\left(\text{a}\text{q}\right)+2{\text{O}\text{H}}^{-}\:\left(\text{a}\text{q}\right)\right]\:+\left[{\text{Z}\text{n}}^{2+}\:\left(\text{a}\text{q}\right)+{2{\text{C}\text{H}}_{3}\text{C}\text{O}\text{O}}^{-}\:\left(\text{a}\text{q}\right)+2{\text{H}}_{2}\text{O}\:\left(\text{l}\right)\right]\to\:{\text{Z}\text{n}\left(\text{O}\text{H}\right)}_{2}\:\left(\text{s}\right)+2{\text{C}\text{H}}_{3}\text{C}\text{O}\text{O}\text{N}\text{a}\:\left(\text{a}\text{q}\right)+2{\text{H}}_{2}\text{O}\:\left(\text{l}\right)$$

(ii) Reaction between NaOH and AgNO_3_

$$\:\left[{2\text{N}\text{a}}^{+}\:\left(\text{a}\text{q}\right)+{2\text{O}\text{H}}^{-}\:\left(\text{a}\text{q}\right)\right]+{2\text{A}\text{g}}^{+}\:\left(\text{a}\text{q}\right)+2{{\text{N}\text{O}}_{3}}^{-}\:\left(\text{a}\text{q}\right)\to\:{2\text{N}\text{a}\text{N}\text{O}}_{3}\:\left(\text{s}\right)+2\text{A}\text{g}\text{O}\text{H}\:\left(\text{s}\right)$$

Step 3: Reaction between zinc and silver hydroxides

$$\:{{\left[\text{Z}\text{n}(\text{O}\text{H}{)}_{4}\right]}^{2-}+\left[2\text{A}\text{g}(\text{O}\text{H}{)}_{4}\right]}^{2-}+\:{4\text{N}\text{a}}^{+}\begin{array}{c}\text{D}\text{W}\\\:\to\:\\\:\text{E}\text{t}\text{h}\text{a}\text{n}\text{o}\text{l}\end{array}\:{\text{A}\text{g}}_{2}\text{O}/\text{Z}\text{n}\text{O}+4\text{N}\text{a}\text{O}\text{H}+2{\text{H}}_{2}\text{O}\:$$

Step 4: Calcination

$$\:{\text{A}\text{g}}_{2}\text{O}/\text{Z}\text{n}\text{O}\:\left(\text{s}\right)\begin{array}{c}500\text{℃}\\\:\to\:\\\:2\:\text{h}\end{array}\text{A}\text{g}/\text{Z}\text{n}\text{O}\:\left(\text{s}\right)+{\text{O}}_{2}\:(\uparrow\:)$$

### Preparation of Electrospun PVDF/ZnO and PVDF/Ag-ZnO NFMs

**Fig. 2:**
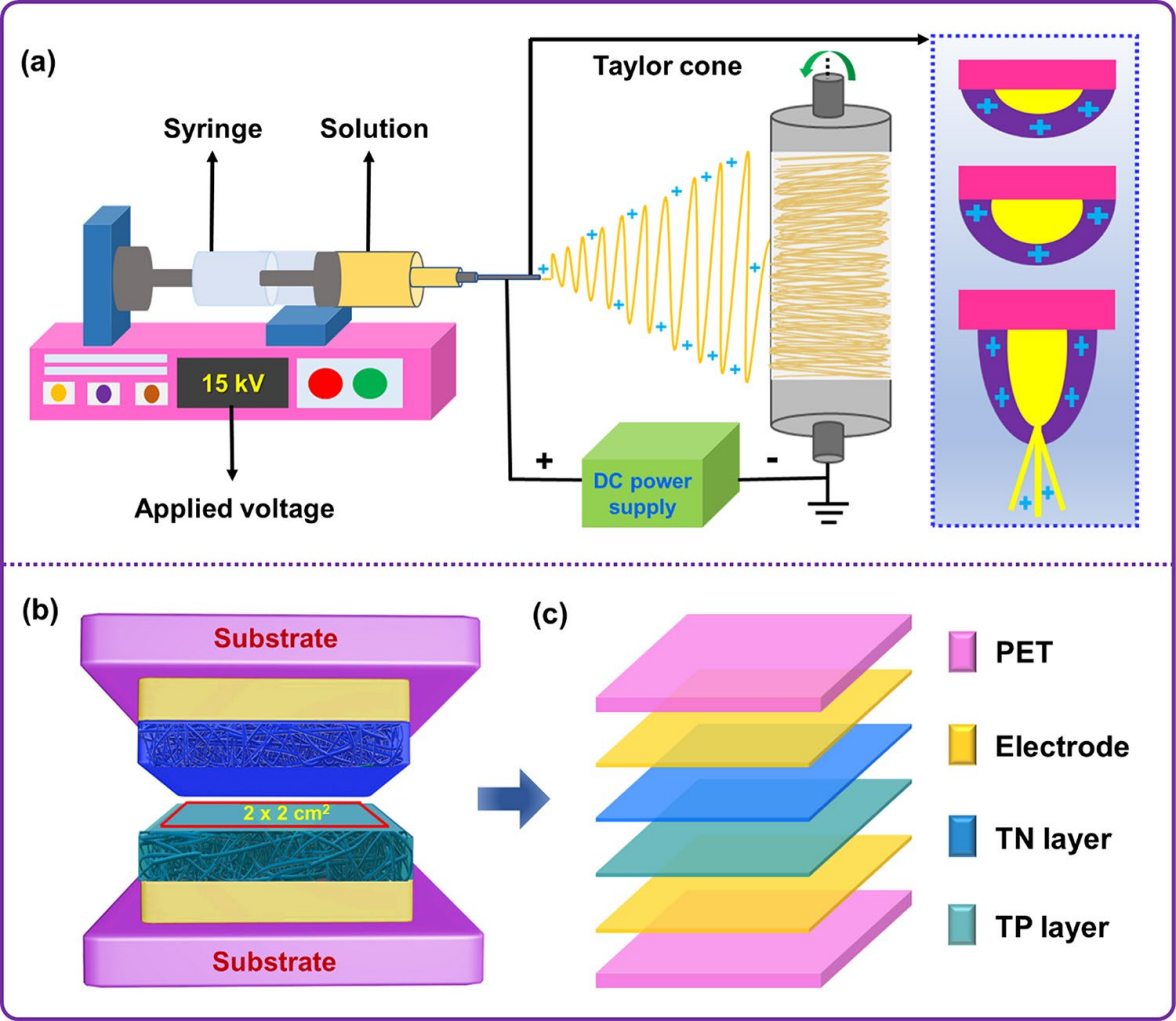
(**a**) Schematic illustration of the electrospinning setup, (**b**) Cross-sectional view of the fabricated TENG device.

The electrospinning of pristine PVDF, PVDF/ZnO (1, 3, and 5 wt%), and PVDF/Ag-ZnO (1, 3, and 5 wt%) were carried out using an electrospinning technique and its setup is shown in Fig. 2a. To prepare the pristine PVDF NFMs (P0), 10 wt% of PVDF powder was dissolved in DMF:acetone solvent mixture (6:4 - v/v), kept at 50 ˚C about 6 h under magnetic stirring at 500 rpm. The resultant homogeneous solution was allowed to cool to ambient temperature and subsequently poured into a 10 mL plastic syringe fitted with a metal needle (23-gauge, an outer diameter of 0.65 mm and an inner diameter of 0.33 mm). Next, the polymer solution-filled plastic syringe was placed on the electrospinning instrument (ESPIN NANO V1-VH, India). The positive end of the high-voltage power supply was linked to the metallic needle tip, while the negative terminal was connected to the drum collector. The electrospinning parameters were configured with an applied voltage of 15 kV using an external DC power supply, a solution feeding rate of 1.2 mL/h, a drum collector rotation speed of 100 rpm, and a tip-to-collector distance of 10 cm. After completing the electrospinning parameters configuration, NFM spun around Teflon-coated PET (polyethylene terephthalate - conductive sheet) which was wrapped around the drum collector. The PET sheet was carefully removed and placed in a vacuum oven at 80 ˚C for 24 h to eliminate the surface residual solvents present over the NFM. The NFM was then cooled to room temperature, cut into the required dimensions and used for the further characterization and experimental studies. Similarly, electrospun PVDF containing ZnO NPs (1, 3, and 5 wt% - coded as PZ1, PZ3, and PZ5, respectively), and Ag-ZnO NPs (1, 3, and 5 wt% - coded as PAZ1, PAZ3, and PAZ5, respectively) NFMs was also fabricated using the aforementioned electrospinning parameters.

### Device fabrication

TENG device in the CS mode was fabricated using TPU as TP layer and different TN layers such as PVDF/ZnO and PVDF/Ag-ZnO based NFMs (P0, PZ1, PZ3, PAZ5, PAZ1, PAZ3 and PAZ5). The TP and TN active layer was made into 2.2 × 2.2 cm^2^ size and affixed to one side of adhesive Ni-Cu coated polyester electrodes (2.0 × 2.0 cm^2^ size). The oversized triboelectric active layers were designed to prevent short circuit between the top and bottom electrodes. Both active layers were then positioned exactly opposite to each other without any gap. Finally, the device was covered with an insulating PET sheet to protect the device from the ambient dust and humidity. Similarly, following the same fabrication procedure, triboelectric active layers measuring 10.2 × 2.2 cm^2^ and Ni-Cu coated polyester electrodes measuring 10.0 × 2.0 cm^2^ were used to fabricate the TENG device for energy harvesting and HCM applications. The device structure and its cross-sectional view is shown in Fig. 2b. The optical images of the fabricated device are shown in the supplementary material Fig. S2.

### Characterizations

Surface morphology and elemental composition of the synthesised NPs and the fabricated electrospun NFMs were analyzed using the Field Emission-Scanning Electron Microscopy (FE-SEM) and Energy-Dispersive X-Ray spectroscopy (EADX, Quanta 250 FEG, USA). Prior to FE-SEM analysis, the samples were gold-coated to enhance conductivity. The diffraction pattern of the synthesised NPs and the fabricated electrospun NFMs were analyzed using X-ray diffraction techniques (XRD, Cu-K_α1_ radiation : λ = 1.5406 Å, Bruker D8 Advanced Diffractometer, Germany). The polymorphic phase changes of PVDF based electrospun NFMs were recorded using Fourier Transform Infrared Spectroscopy (FT-IR, Shimadzu IR Affinity-1, Japan). X-ray photoelectron spectroscopy (XPS) measurements were carried out for the NPs chemical compositions and its state (Source: Al-*K*_α_, thermo scientific, US). The photoluminescence (PL) spectra of NPs were measured using fluorescence spectrometer (Model: Hitachi-F7000, Hitachi high-tech corporation, Japan). The wettability properties of the NFMs were tested using the Water Contact Angle (WCA) measurements (Method - Sessile drop : 1 µL - droplet volume of water, DSA25B - Kruss Advance Instrument, Germany). Atomic Force Microscopy (AFM, Model: Nanosurf AFM, Switzerland) was used for analyzing the surface topography and roughness of the samples. The dielectric measurements were carried out using an impedance analyzer (Model: PSM 1735, Newton’s 4th limited, UK). The surface potentials of the tribonegative layers were measured using the Ti/Ir tip non-contact mode of Scanning Kelvin Probe Microscopy (SKPM, Model: ASYELEC-02-R2, Oxford Instruments). The electrical properties like open circuit potential (*V*_oc_) of the TENG devices were tested using a digital phosphor oscilloscope (*R*_in_ = 40 MΩ, Tektronix - DPO4104, USA). Also, the short circuit current (*I*_sc_) was tested in the similar setup, with the oscilloscope was linked to a low-noise current pre-amplifier (SR570, Stanford Research Systems, USA). The NI-DAQ (BIOPAC - MP150, USA) system connected to a PC with (LabVIEW program - Acknowledge 4.2 Software ) was used to acquire real-time data for energy harvesting applications and different biomechanical movements in HCM measurements. All measurements were performed at standard room temperature conditions.

## Results and discussion

**Fig. 3:**
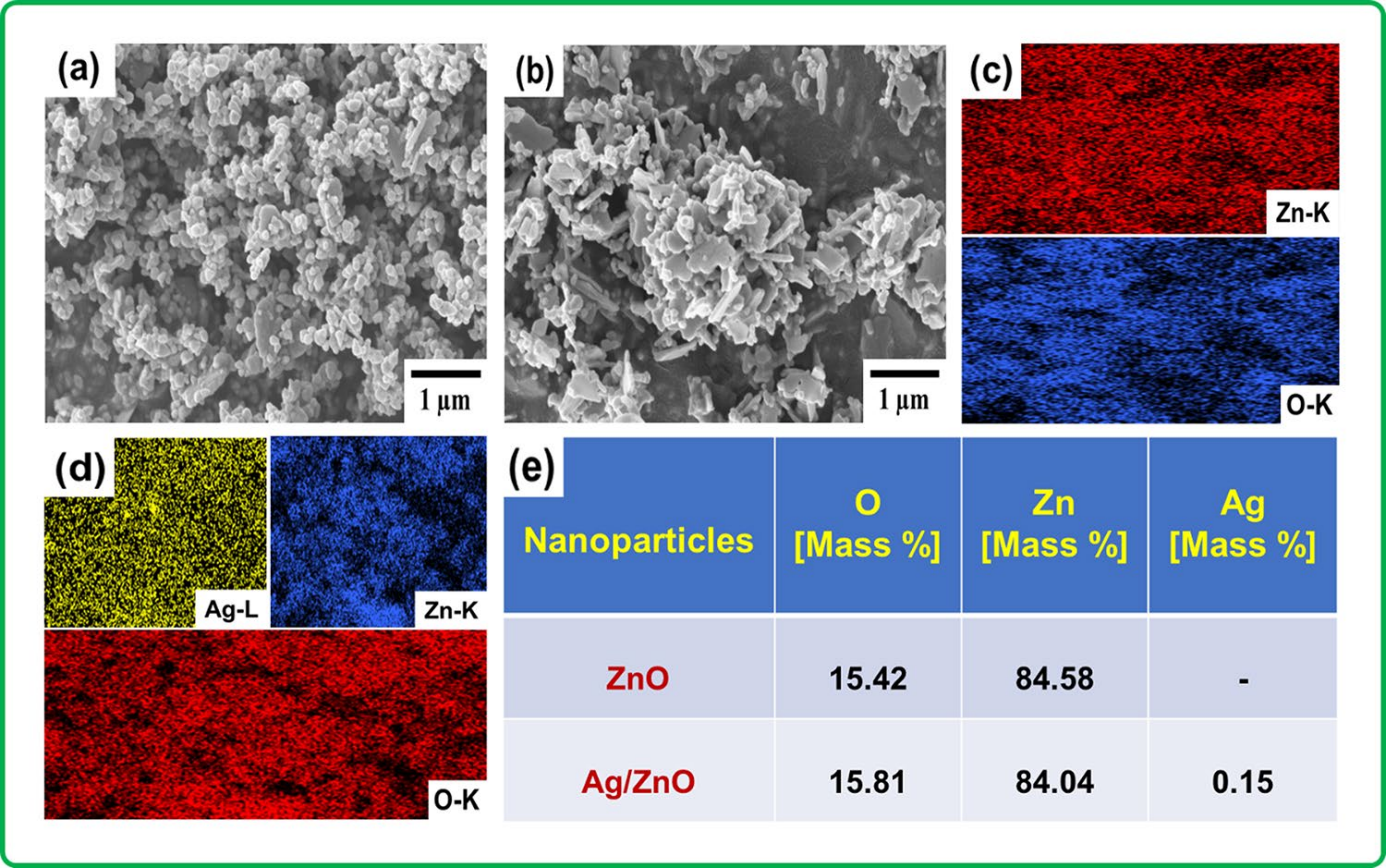
(**a**) SEM micrographs of ZnO NPs, (**b**) SEM micrographs of Ag-ZnO NPs, (**c** and **d**) EDAX elemental mappings of ZnO, Ag-ZnO NPs, respectively, (**e**) Elemental compositions of the as-synthesised ZnO and Ag-ZnO NPs.

**Fig. 4:**
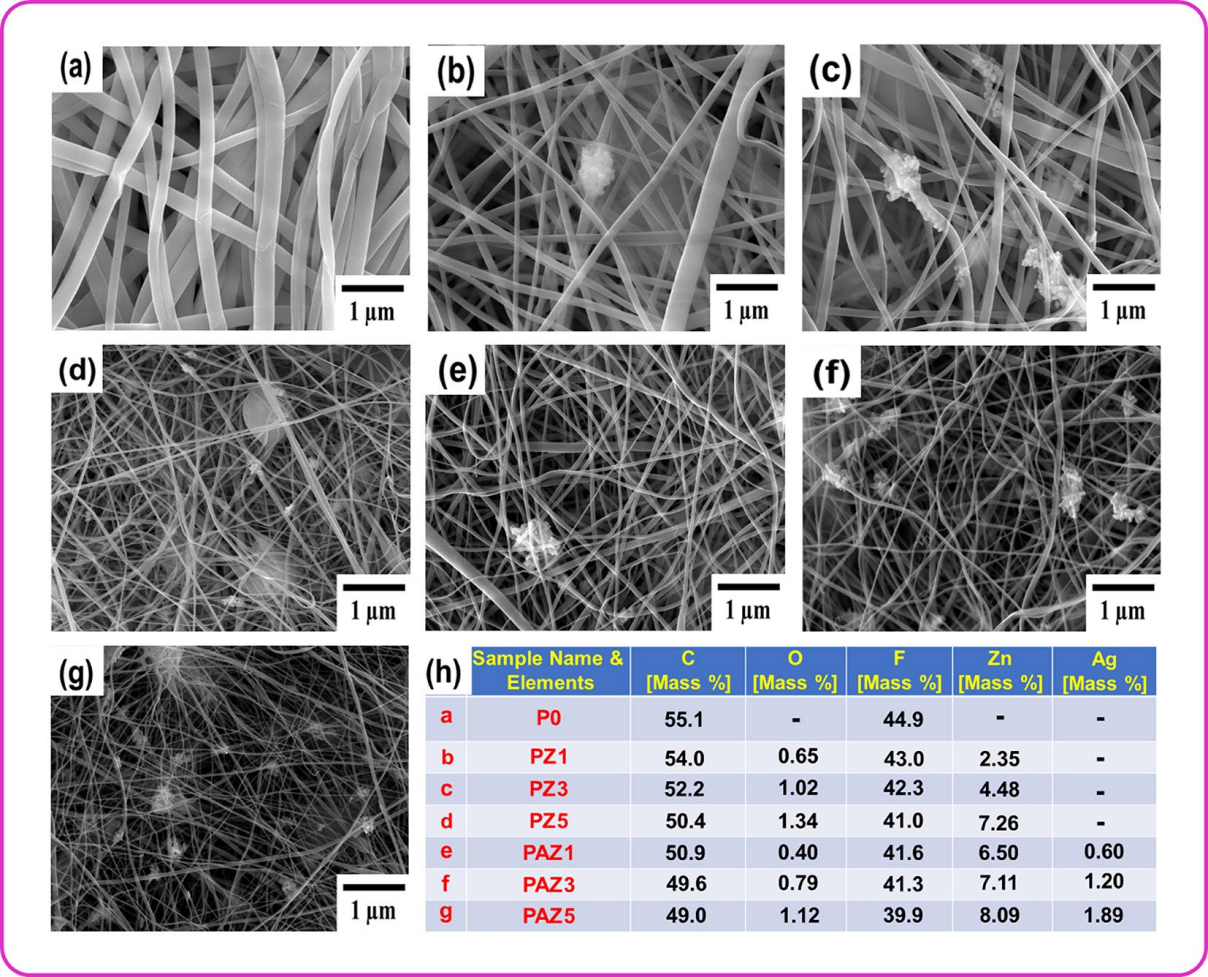
(**a**–**g**) SEM micrographs of the electrospun NFs: P0 (Pristine PVDF), PZ1, PZ3, PZ5 (with increasing ZnO wt%), PAZ1, PAZ3, and PAZ5 (with increasing Ag-ZnO wt%), respectively, (**h**) Elemental compositions analysis of the NFs: P0, PZ1, PZ3, PZ5, PAZ1, PAZ3, and PAZ5.

SEM micrographs were used to investigate surface morphology and elemental analysis of synthesized ZnO and Ag-ZnO NPs. As shown in Fig. 3a. and b, SEM micrographs of ZnO, and Ag-ZnO NPs revealed spherical and plate-like shapes, respectively. Elemental analysis confirmed the chemical composition and purity of the synthesized ZnO and Ag-ZnO NPs as shown in Fig. 3c. and d. Additionally, Fig. 3e. provides a detailed elemental composition of ZnO, and Ag-ZnO NPs. The SEM micrographs of fabricated electrospun NFMs such as P0, PZ1, PZ3, PZ5, PAZ1, PAZ3, and PAZ5 were shown in Fig. 4a-g, respectively. The impact of ZnO and Ag-ZnO NPs doping on the fiber arrangement of electrospun PVDF was examined using ImageJ software. Measurements were taken from 51 different locations to determine the fiber average diameter, which ranged between 212 and 103 nm. The distribution curves and corresponding histograms were given in Fig. S3. in the supplementary material. It was observed that the average fiber diameter decreased with increasing NPs doping wt%. Several factors, including the kinetic energies of the elongating jet, solvent evaporation rate, viscosity, electrical forces, and voltage, play significant roles in determining the NFMs diameter. The addition of metallic ZnO, and Ag-ZnO NPs increases the conductivity, leading to an improved charge density on the surface jet, which consequently increasing the elongating force and jet velocities. Consequently, the enhanced conductivity of the electrospinning solution resulted in thinner fiber formation, affecting factors like diameter and alignment^22,28^. The agglomerated NPs (regions rich in NPs) form connecting networks that act as pathways for charge transfer across the electrospun PVDF matrix. These networks provide additional contact points between the PVDF polymer chains and NPs surfaces, leading to stronger interfacial interactions^22^. SEM and EDAX mapping (Fig. S4. in the supplementary material) also revealed that Ag-ZnO NPs are embedded within the fiber matrix was even, where they introduce heterojunctions at the interface, further supporting interfacial charge separation and transfer to the surrounding PVDF chains. This effect is useful for enhancing the TENG performance, as it increases the dielectric constant of the composite, thereby enhancing charge generation and improving the electrical output^29^. The EDAX spectrum is given in Fig. S5. in the supplementary material, shows that the P0 sample contained only the C (55.1%) and F(44.9%) elements and PAZ3 sample contained the elements C (49.6%), F (41.3%), O (0.79%), Zn (7.11%), and Ag (1.20%), respectively. Figure 4h. provides a detailed elemental composition all the fabricated NFMs. These findings confirmed that the ZnO, and Ag-ZnO incorporated PVDF was free from external impurities. However, bead formation observed in PZ5 and PAZ5 samples is likely due to higher conductivity within nanofibers. SEM micrographs of electrospun P0, PZ1, PZ3, PZ5, PAZ1, PAZ3, and PAZ5 NFMs demonstrated an uneven fibrous morphology without any external impurities. Among the analyzed combinations, P0, and PAZ3 were identified as the most optimized and were selected for further TENG device fabrication and electrical studies. Additionally, to better understand the polymorphic phase changes, the fabricated nanofibers were characterized by using XRD and FT-IR.

**Fig. 5:**
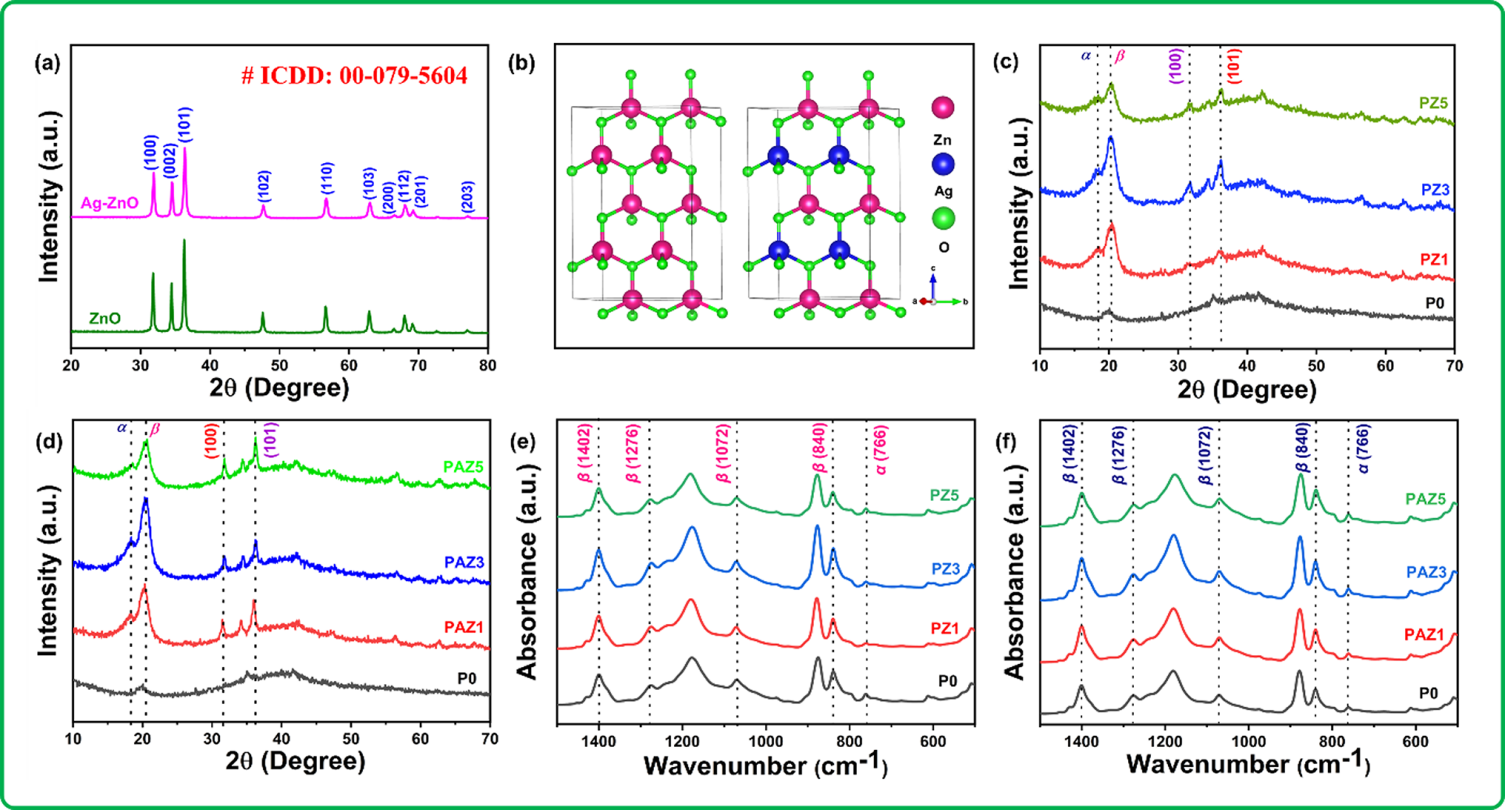
(**a**) XRD patterns of ZnO and Ag-ZnO NPs, (**b**) Ball and stick model illustrating crystalline structure of ZnO and Ag-ZnO NPs, (**c**) XRD patterns of NF samples P0, PZ1, PZ3, PZ5, (**d**) XRD patterns of NF samples PAZ1, PAZ3, and PAZ5, (**e**) FT-IR spectra of NF samples: P0, PZ1, PZ3, PZ5, showing the effect of ZnO content, (**f**) FT-IR spectra of NF samples: PAZ1, PAZ3, and PAZ5, illustrating the effect of Ag-ZnO in PVDF.

The crystallographic properties of the synthesized ZnO and Ag-ZnO NPs were analyzed using an X-ray diffractometer. The XRD pattern for the synthesized Ag-ZnO NPs delivered diffraction peaks at the 2θ positions of 31.92˚, 34.61˚, 36.46˚, 47.73˚, 56.77˚, 62.94˚, 66.59˚, 68.10˚, 69.20˚ and 77.15˚, corresponding to the crystal planes (100), (002), (101), (102), (120), (103), (200), (112), (201), and (202), respectively as shown in Fig. 5a. The ball and stick model of the crystal structure for ZnO and Ag-ZnO NPs is depicted in Fig. 5b. These peaks were compared to the reference card no. 01-079-5604 from the International Centre for Diffraction Data (ICDD), confirming that the synthesized NPs exhibits a hexagonal wurtzite structure (*α* = *β* = 90˚; *γ* = 120˚, a = b = 3.249 Å; c = 5.205 Å) with the PC3mc (186) space group. The precise alignment of all indexed peaks at 2θ angles signified the structural stability upon Ag doping at Zn sites, consistent with previous studies^23^. The absence of additional peaks in the XRD patterns highlighted the phase purity of all compositions. The narrow and sharp diffraction peaks signified the crystalline nature of the synthesised NPs. The crystallite size was calculated using Scherrer’s Eq. (1) based on the most intense peak plane (101).

1 $$\:D=\frac{K{\uplambda\:}}{\beta\:\text{c}\text{o}\text{s}\text{ϴ}}$$

Here, *K* denotes the Scherrer’s constant (K = 0.9), λ is the wavelength of Cu-K_α1_ radiation (λ = 1.5406 Å), *β* represents the full width at half maxima (FWHM) of the diffraction peak (101), and θ is the Bragg’s angle. The calculated value of ‘*D*’ was 33.24 nm. Figure 5c. and d. represents the XRD patterns of pristine PVDF and NPs doped PVDF. The P0 sample exhibited characteristic peaks of PVDF, specifically the *α-*phase at 18.2˚ (100) and *β-* phase at 20.4˚ (110). In contrast, the samples with ZnO and Ag-ZnO NPs wt% doping displayed 2θ values for both the doped NPs and PVDF. Additionally, the peak intensities of the *α-*phase (18.2˚) decreased, while those of the *β-*phase (20.4˚) increased, which is indicating the addition of ZnO and Ag-ZnO NPs facilitated phase transformation in PVDF. The increasing in the *β*-phase may be due to the effective nucleation between the PVDF and NPs, while the decrease trend in the *β*-phase may be due to high NPs concentration in the polymer chain matrix, hindering nucleation between PVDF and NPs^23^. Therefore, the XRD patterns indicate that the NPs doped into the PVDF matrix and maintain their hexagonal crystal structure. The concurrent presence of PVDF and ZnO and Ag-ZnO NPs peaks in the XRD diffractograms further confirm the formation of composites labelled PZ1, PZ3, PZ5, PAZ1, PAZ3, and PAZ5.

FT-IR spectra were utilized to differentiate between the electroactive and electro-inactive polymeric phases of pristine PVDF and PVDF doped with NPs. Distinct peaks at 1172 and 1402 cm^− 1^correspond to CF_2_ and CH_2_ stretching and vibrations, respectively. Peaks observed at 766, 795, 875, 972, and 1172 cm^− 1^ are accounting to the *α*-phase, where as those at 840, 1072, 1276, and 1402 cm^− 1^ are attributed with the *β*-phase^30^. The most prominent peaks are labeled accordingly in the Fig. 5e. and f. It is noteworthy that the intensities of *α*- and *β*- phases varied with the doping wt% of different NPs such as ZnO and Ag-ZnO NPs in PVDF, indicating changes in the PVDF polymorphic phases. This spectrum allowed for the determination of the *β*-phases fraction within the fabricated NFMs. Using Lambert-Beer’s law, the *β*-phase fraction F(*β*) related to the *α*-phase can be calculated^31^.

2 $$\:F\left(\beta\:\right)=\frac{{A}_{\beta\:}}{(1.26\times\:{A}_{\alpha\:})+{A}_{\beta\:}}\times\:100\:\%$$

Where *A*_α_ and *A*_β_ represents the absorption fractions of the *α-* and *β-* phases at 766 and 840 cm^− 1^ respectively. The constant 1.26 indicates the ratio of the absorption co-efficient of the *β-*phase (7.7 $\:\times\:$ 10^4^ cm^2^ mol^− 1^) and *α-*phase (6.1 $\:\times\:$ 10^4^ cm^2^ mol^− 1^).

**Fig. 6:**
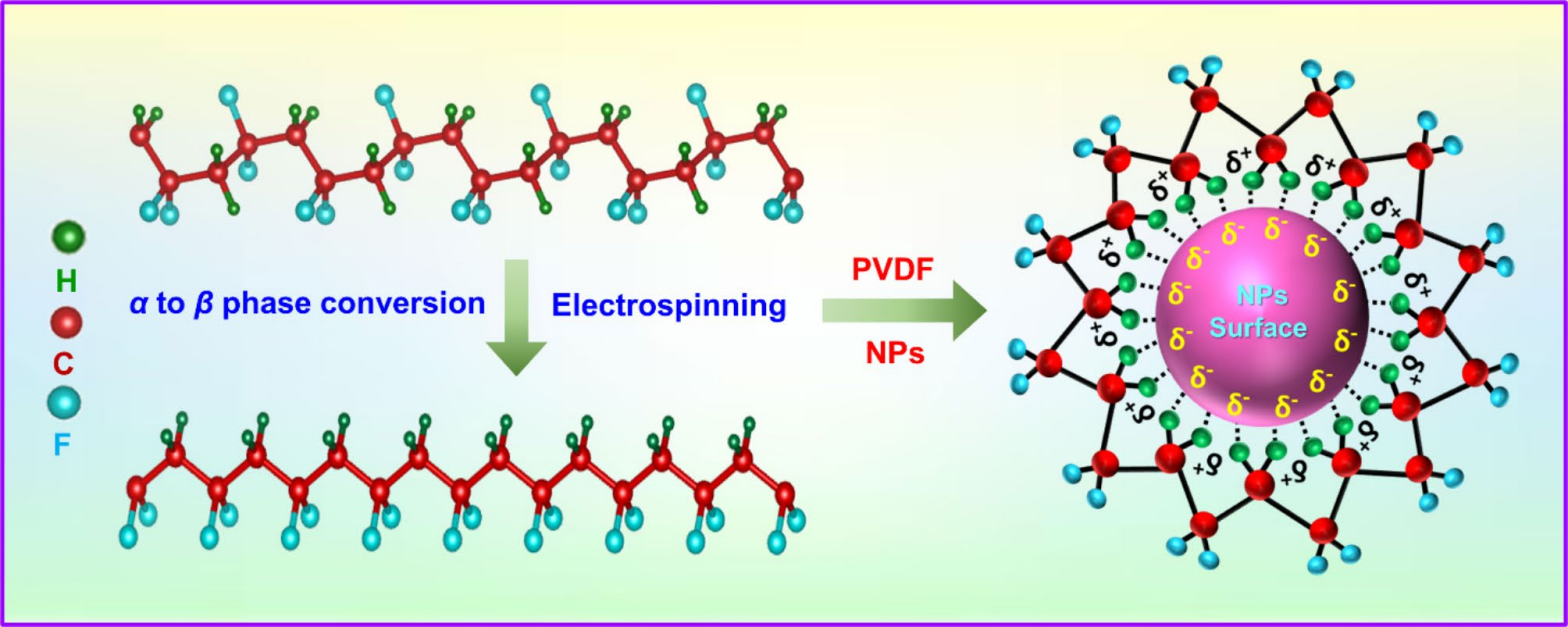
Schematic illustration depicting the interaction mechanism between PVDF and NPs.

Using Eq. (2), the *β*-phase fractions of P0, PZ1, PZ3, PZ5, PAZ1, PAZ3, and PAZ5 were calculated as 64%, 79%, 82%, 76%, 83%, 88% and 81%, respectively. Correspondingly, the calculation is given in the supplementary material Table S2. The calculation revealed that PAZ3 exhibits the high *β*-phase %. The optimal concentration of Ag-ZnO NPs was determined to be 3 wt% for the PVDF matrix, likely owing to the higher interfacial interactions between the Ag-ZnO NPs (3 wt%) and PVDF. This phenomenon can be accounted by the ion-dipole interaction mechanism shown in Fig. 6. In PVDF, the electronegativity of carbon (C) lies between that of hydrogen (H) and fluorine (F). As a result, within the PVDF chain F atoms having higher electron affinity (partial negative charge) than H (with partial positive charge). The partial positive H atoms in PVDF interact with Ag-ZnO NPs through ion-dipole interactions, where Ag-ZnO NPs serves as a nucleating agent in PVDF^23,32^. Consequently, the PVDF chain align on the surface of Ag-ZnO NPs, forming a stable *β*-phase (all-trans : *TTTT*) in PVDF. This interaction promotes the orientation of PVDF in the polymer, contributing to the increased *β*-phase. This interaction drives the *β*-phase nucleation with up to 3 wt% of Ag-ZnO NPs added to the PVDF matrix. However, when the Ag-ZnO NPs content exceeded 3 wt%, the *β*-phase fraction decreases. Excessive Ag-ZnO NPs cause agglomeration on the PVDF surface, hindering effective interfacial interaction and PVDF molecular chains from aligning properly, resulting in a reduced *β*-phase. Since H in PVDF interacts with Ag-ZnO NPs, the outward projected -CF_2_- groups enhance the triboelectric performance by improving the tribonegativity of the NFs and minimizing triboelectric loss. Therefore, the increased *β*-phase fraction of PAZ3 NFMs makes it a potential triboelectric layer for fabricating TENG devices and efficient in real-time applications.

XPS is a highly important technique used to investigate the surface chemical composition and states of materials. The obtained XPS spectrum for ZnO and Ag-ZNO NPs is given in the supplementary material Fig. S6. The results show the binding energies for Ag doped 1045.5 eV for Zn 2p_1/2_, 1022.4 eV for Zn 2p_3/2_, 532.1 eV for O 1s, 374.6 eV for Ag 3d_3/2_, 368.4 eV for Ag 3d_5/2_. The difference between the peaks of Zn 2p_1/2_ and Zn 2p_3/2_ is 23.1 eV, which indicates that the Zn is present in + 2 oxidation state. Similarly, the difference between the peaks of Ag 3d_3/2_ and Ag 3d_5/2_ is 6.2 eV, which indicates that the Ag is present in + 1 oxidation state. This, findings confirms that Ag^+^ ions are successfully doped into the ZnO lattice. Doping of Ag^+^ into Zn^+ 2^ ions lead to a charge imbalance in the lattice that influences the electronic properties of ZnO. This doping is useful for enhancing the performance of TENG, as the charge imbalance enhances the polarization within the ZnO structure^33,34^.

PL spectra of ZnO and Ag-ZnO NPs were measured using an excitation wavelength of 350 nm in the room temperature. The PL plot for ZnO and Ag-ZnO NPs is given in the supplementary material Fig. S7. ZnO NPs show a near band edge emission peak centered at 420 nm, with no additional emission peak in the visible region. In contrast, Ag-ZnO NPs exhibit an emission peak at 397 nm with significantly reduced intensity. This noticeable quenching of the PL emission in Ag-ZnO is attributed to efficient interfacial charge transfer from ZnO to Ag, which reduces the radiative recombination. This interfacial charge transfer resulting from Ag doping is advantageous for TENG performance. By reducing radiative recombination, more free charges become available within the ZnO lattice, which can enhance surface charge density, dielectric properties, and boost overall electron transfer in the TENG device^33,34^.

WCA is a measure of surface wettability, which is influenced by the surface energy and roughness. In this investigation, we used the sessile drop technique with 1 µL water droplets to evaluate the WCA of the ZnO and Ag-ZnO doped PVDF NFMs. The WCA values for P0, PZ3, and PAZ3 was measured as 94°, 117°, and 123°, respectively. The WCA values for the NFMs samples are shown in Fig. S8. in the supplementary material. It was noted that all TN layers exhibited a consistent enhancement in hydrophobicity. These results align with the Wenzel model, which posits that roughening an inherently hydrophobic surface increases the WCA, signifying greater hydrophobicity. The WCA data analysis indicates that doping of NPs into the PVDF polymer matrix enhances the hydrophobic nature of the TN layer^28^.

The surface roughness of PVDF and its composite NFs was analyzed using AFM as shown in Fig. S9. in the supplementary material. According to the topographical results, P0 has an average surface roughness of 47 nm and an average valley depth of 351 nm. In contrast, PZ3 exhibit an increased average surface roughness of 53 nm, which further rises in the PAZ3 sample to a maximum of 62 nm. Similarly, the valley depths increase from 448 nm for PZ3 to 625 nm for PAZ3. The presence of these hill-and-valley structures in the P0, PZ3, and PAZ3 samples is beneficial for TENG performance enhancement, as it improves frictional interaction across the triboelectric layers. The corresponding topographical parameters were given in the supplementary material Table S3. Further, the AFM data effectively supports the hydrophobic nature of P0, PZ3, and PAZ3 samples by showing that the increased surface roughness and valley depth, combined with the exposure of -CF_2_ groups are the key factors in enhancing hydrophobic nature. This hydrophobicity is advantageous for TENG devices, as it protects against environmental humidity, minimizes charge dissipation, and ultimately contributes to higher charge density in the triboelectric layer^13^.

Dielectric measurements were conducted for P0, PZ3, and PAZ3 samples, with dielectric constant and dielectric loss recorded under ambient conditions (25 °C and 47% relative humidity). As shown in Fig. S10(a) in the supplementary material. P0 exhibited a dielectric constant of 1.78 at 100 Hz. This value gradually increased with the addition of ZnO and Ag-ZnO to PVDF, reaching 11.49 for PZ3 and 37.68 for PAZ3, and is attributed to the expansion of the interfacial area and enhanced charge trapping capacity. Similarly, Fig. S10(b) in the supplementary material shows that these NFs maintain a relatively low dielectric loss, with values of 0.18 for P0, 0.91 for PZ3, and 1.68 for PAZ3 at 100 Hz. This also indicates efficient charge-holding ability and minimal charge dissipation. Based on these results, we conclude that incorporating ZnO and Ag-ZnO NPs into PVDF enhances its dielectric constant, and contributes towards improving overall TENG performance^22^.

SKPM was used to quantitatively characterize the surface potentials of electrospun NFs at the nanoscale. The surface potential profiles of the TN layers are shown in Fig. S11. in the supplementary material. The individual NFs of P0, PZ3, and PAZ3 exhibited negative potentials of -60 mV, -115 mV, and − 170 mV, respectively. The data indicate that the addition of ZnO and Ag-ZnO NPs into PVDF shifts the surface potential of PVDF to more negative values, with P0 increasing from − 60 mV to a higher negative potential of -170 mV. This increase in surface potential can be attributed to enhanced interfacial interaction between the NPs and PVDF^35,36^.

Charge density is key parameter that directly influences the electrical performance of TENGs. The triboelectric charges generated during material contact and separation are closely linked to charge density, impacting the efficiency and effectiveness of the energy conversion process. As the filler in the PVDF matric changes, the total interfacial area per unit volume also increases. This enhancing the average electrical polarization associated with each NPs and the coupling between PVDF matrix. This effect leads to an improved dielectric constant in the NFs, thereby boosting their electrical performance^9,37^. Correspondingly, the surface charge density follows the same trend as the dielectric constant. The charge density values for P0, PZ3, and PAZ3 are 6.03, 13.3, and 18.8 µC/cm^2^, respectively. Fig. S12. in the supplementary material shows the charge density plot.

### Working mechanism

**Fig. 7:**
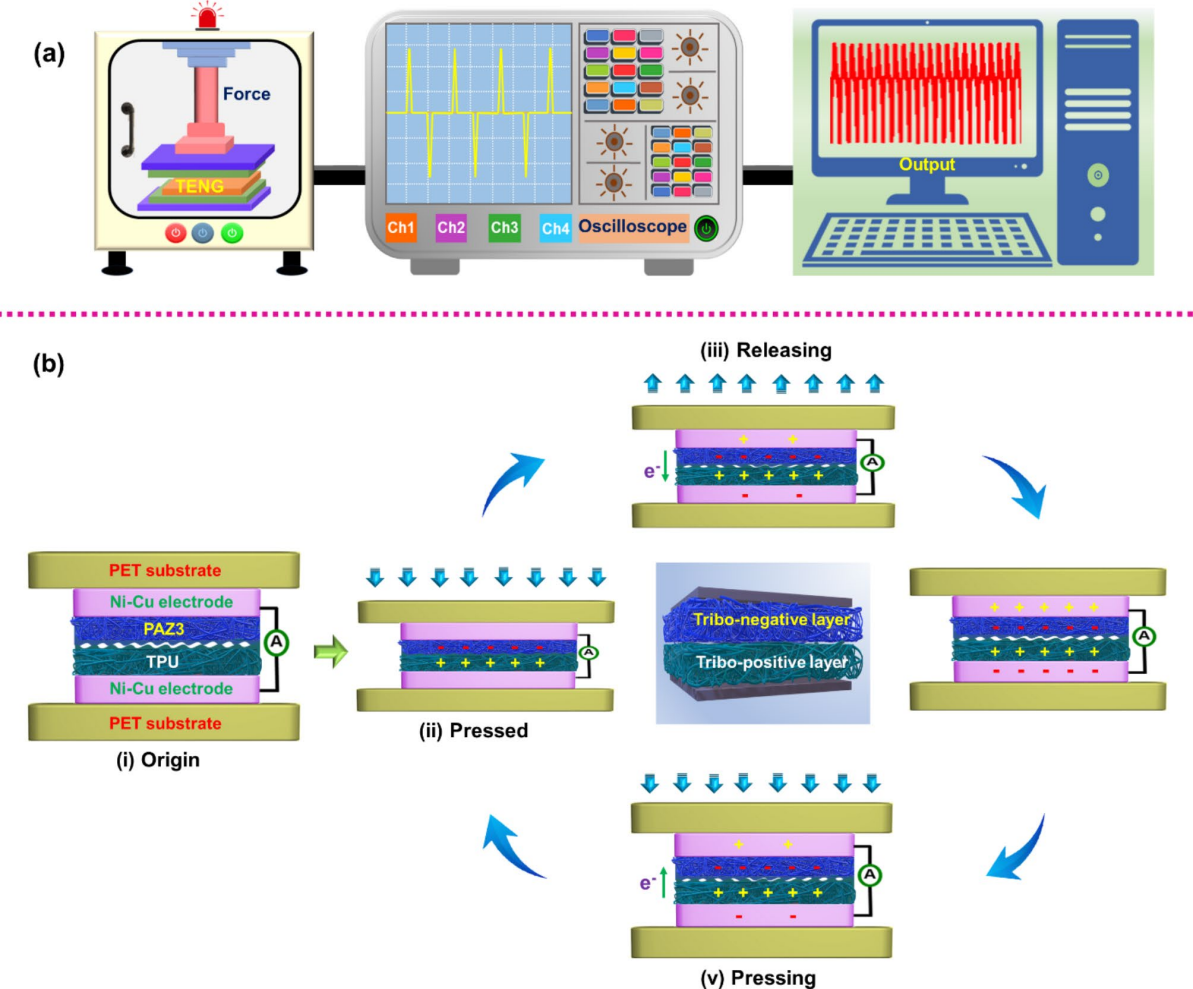
(**a**) Schematic illustration of the instrument setup used for the measurement of the fabricated TENG devices, (**b**) Working mechanism of the PAZ3/TPU-TENG, highlighting the triboelectric interaction and charge generation process.

In this investigation, the fabricated PAZ3/TPU TENG device operates under CS mode. Fundamentally, CS working mechanism interplay between the contact electrification (triboelectrification) and electrostatic induction^13^. According to the triboelectric series, TPU was used as the TP layer and PAZ3 used as the TN layer to fabricate the TENG. As depicted in the original state TEL exhibit a partial spacing before applying the external force. This partial spacing is due to the uneven surface of the TPU layer and PAZ3 layer. According to the Volta-Helmholtz theory, the uneven surface of the NFMs is crucial for improving contact area across the triboelectric layers. Consequently, the improved contact area between the triboelectric layers leads to the higher electrical performance of TENG. The instrument setup and its working mechanism is depicted in Fig. 7a. and b, respectively. While the outside force is applied, the TP and TN compress together, and developing maximum contact. Owing to the maximum contact, the TELs undergoes triboelectrification, where the TP layer acquires maximum positive charge and the TN layer acquires maximum negative charge. When the external force is removed, TELs were separate partially. This partial separation induces a positive charge over Ni-Cu electrode attached to the TN layer and negative charge over Ni-Cu electrode attached to the TP layer through electrostatic induction. Due to unbalanced charges over the TELs and Ni-Cu electrodes, a triboelectric potential difference occurs. Owing to this potential difference, electron flow from bottom electrode to top electrode, initiating the first positive half-cycle of AC signal. The electron flow continues until an equilibrium state is reached. When the outside force is applied again, the electron flow reverses from top to bottom electrode, generating the negative second half-cycle of the AC signal. This cyclic process repeats multiple times during periodic pressing and releasing, producing a steady and continuous electrical output from the TENG device.

### Optimization of TENG electrical performance

**Fig. 8:**
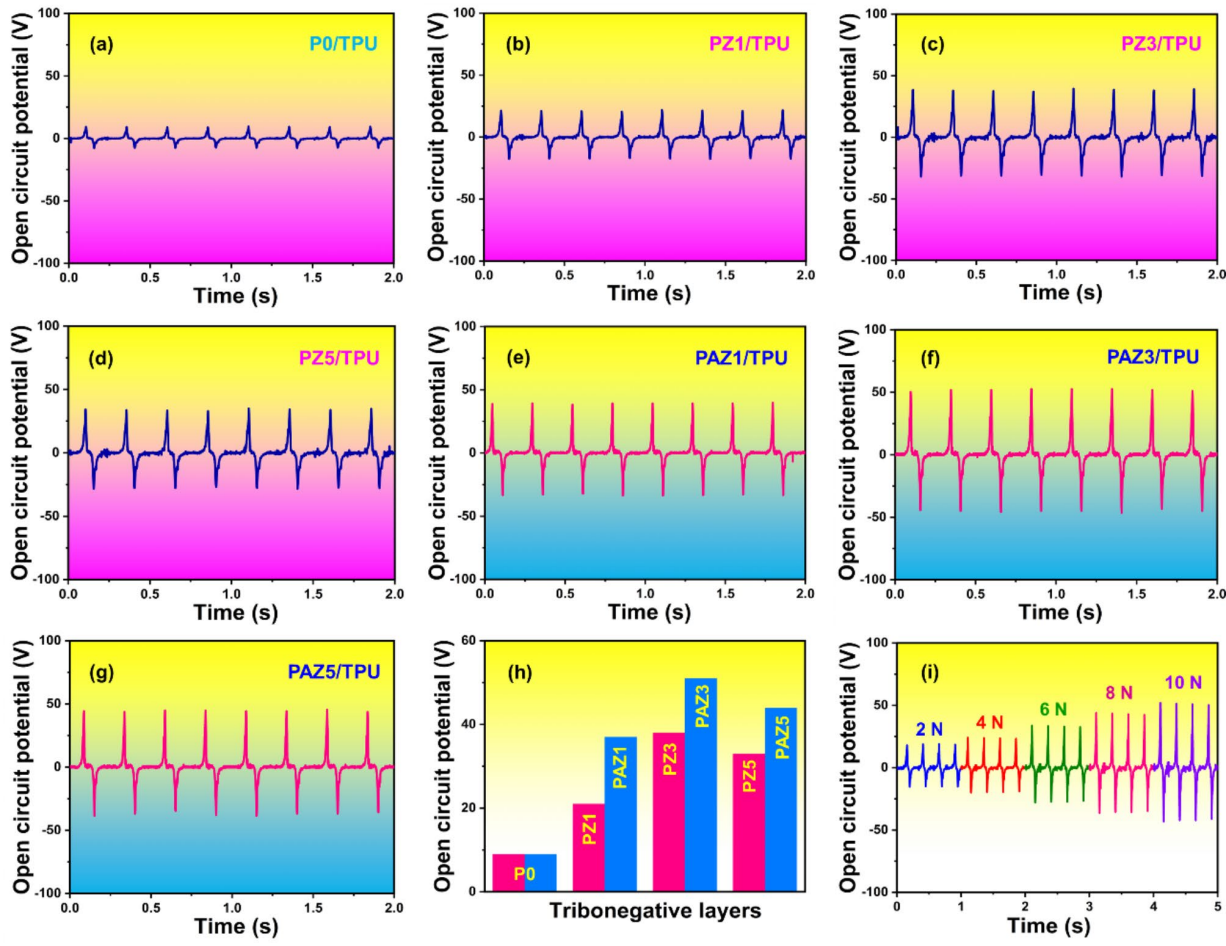
(**a**–**g**) Open circuit potential (*V*_oc_) of TPU-based triboelectric pairs with: P0, PZ1, PZ3, PZ5, PAZ1, PAZ3, and PAZ5, respectively, (**h**) Quantitative plot showing the *V*_oc_ values of P0, PZ1, PZ3, PZ5, PAZ1, PAZ3, and PAZ5 for comparison, (**i**) *V*_oc_ of the optimized PAZ3/TPU-TENG under various load conditions (2 N to 10 N), demonstrating the device performance across different external load.

**Fig. 9:**
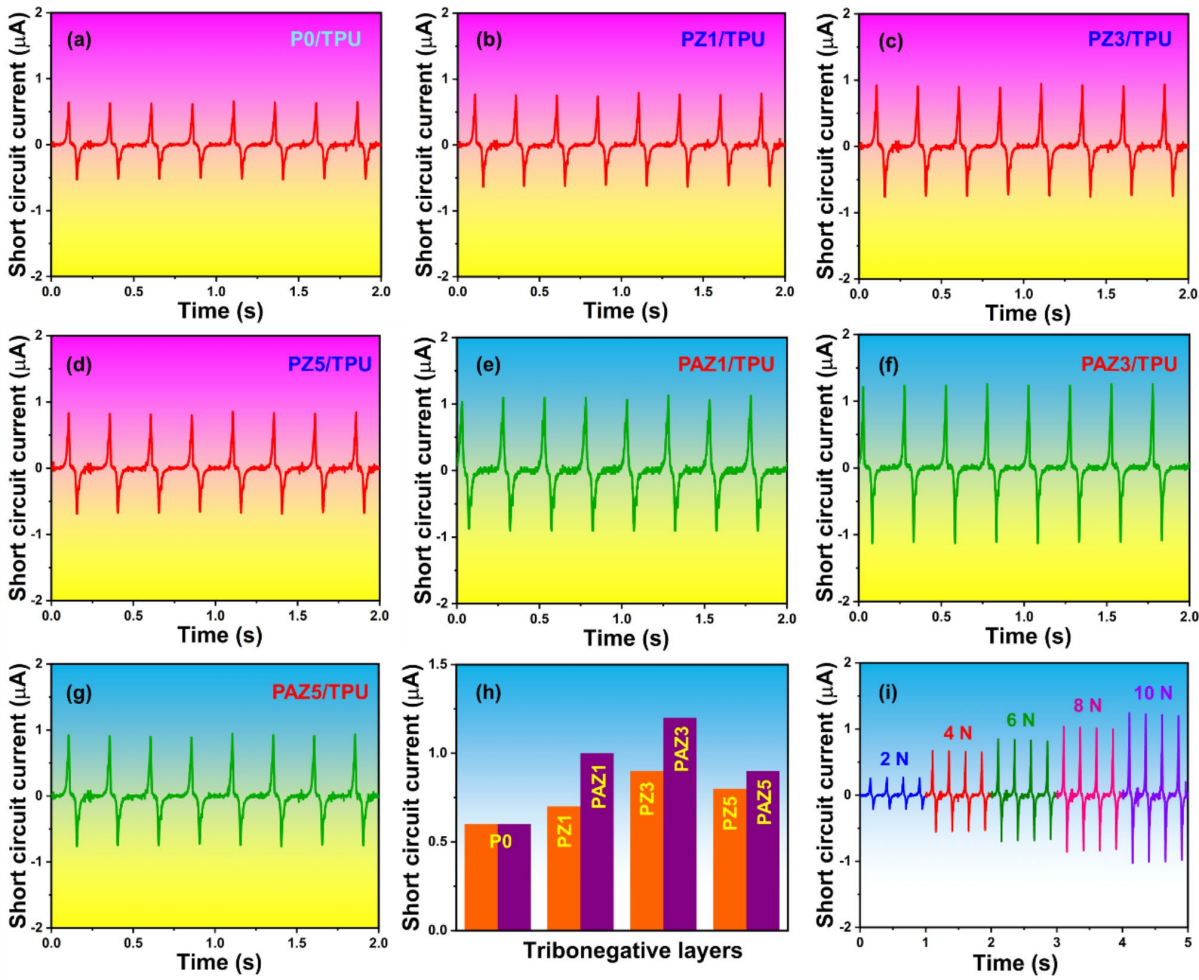
(**a**–**g**) Short circuit current (*I*_sc_) of TPU-based triboelectric pairs with: P0, PZ1, PZ3, PZ5, PAZ1, PAZ3, and PAZ5, respectively, (**h**) Quantitative plot for the *I*_sc_ of P0, PZ1, PZ3, PZ5, PAZ1, PAZ3, and PAZ5 for comparison, (**i**) *I*_sc_ of the optimized PAZ3/TPU-TENG under various load conditions (2 N to 10 N), demonstrating the device performance across different external load. .

The optimization of device output performance, including *V*_oc_, and *I*_sc_ evaluations were conducted on 2 $\:\times\:$ 2 cm^2^ TENG device under a periodic load (10 N, f = 4 Hz). Initially, the electrical performance was assessed using TPU as the TP layer and changing the TN layers (P0, PZ1, PZ3, PAZ1, PAZ3, and PAZ5). The *V*_oc_ for TPU- P0, PZ1, PZ3, PAZ1, PAZ3, and PAZ5 based triboelectric pairs were 9.0 V, 21 V, 38 V, 33 V, 37 V, 51 V, and 44 V respectively, and it is shown in Fig. 8a-g. The quantitative plot for the *V*_oc_ of P0, PZ1, PZ3, PZ5, PAZ1, PAZ3, and PAZ5 is presented in Fig. 8h. and the *V*_oc_ of PAZ3/TPU-TENG under various load condition (2 N to 10 N) is presented in Fig. 8i. Similarly, the *I*_sc_ values were observed as 0.6 µA, 0.7 µA, 0.9 µA, 0.8 µA, 1.0 µA, 1.2 µA, and 0.9 µA respectively, as shown in Fig. 9a-g. The quantitative plot for the *I*_sc_ of P0, PZ1, PZ3, PZ5, PAZ1, PAZ3, and PAZ5 is presented in Fig. 9h. and the *I*_sc_ of PAZ3/TPU-TENG under various load condition (2 N to 10 N) is presented in Fig. 9i. The electrical output (*V*_oc_, *I*_sc_) displayed an increasing trend with varying the NPs doping. When the ZnO and Ag-ZnO NPs doping increases from 0 to 3 wt%, the *V*_oc_ of the TENG device steadily increased (ZnO: from 21 V to 38 V and Ag-ZnO: from 37 V to 51 V). This performance enhancement is attributed to the charge-trapping ability of ZnO and Ag-ZnO NPs. The accumulated static charge at the solid interface enhances the triboelectric surface, thus improving TENG performance. However, beyond 3 wt%, the *V*_oc_ of the TENG device started decreasing (ZnO: from 38 V to 33 V and Ag-ZnO: from 51 V to 44 V). The decrease in electrical output is due the agglomeration, which leads to the formation of interconnecting networks within the electrospun NFMs^22^. Based on the electrical optimization, the PAZ3/TPU-based TENG device demonstrated superior electrical performance compared to other devices, making optimal choice for the further energy harvesting and HCM applications.

### Effect of external load resistance on PAZ3/TPU-TENG

**Fig. 10:**
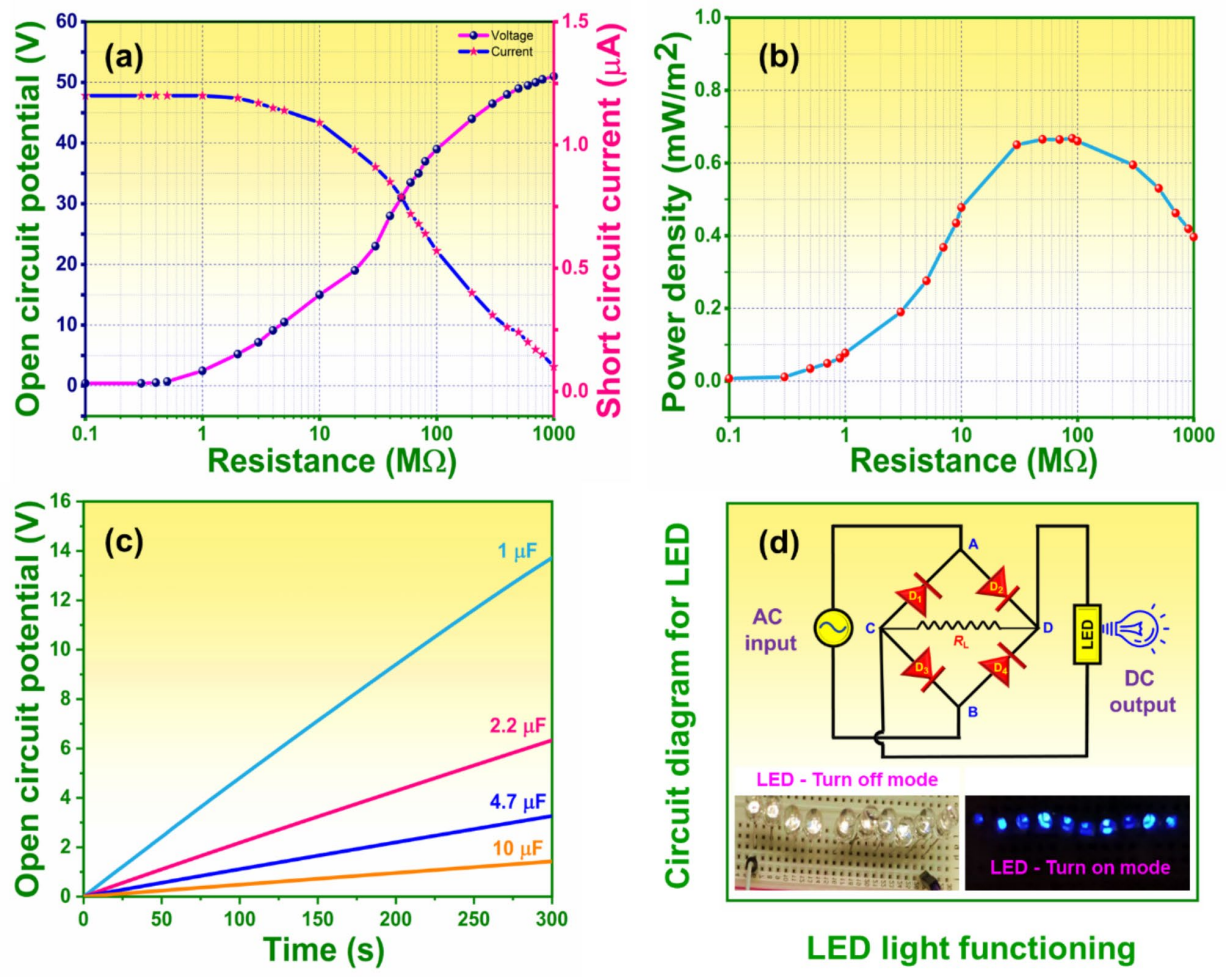
Electrical performance of the optimized PAZ3/TPU-TENG device: (**a** and **b**) Impact of the external load ranging from 0.1 MΩ to 1.0 GΩ on the triboelectric performance and its power density curve, respectively, (**c**) Capacitor charging performance with capacitance ranging from 1.0 µF to 10 µF, (**d**) Circuit diagram illustrating the connection of LEDs to the PAZ3/TPU-TENG device, along with optical images of 10 LEDs before and after being powered by the device.

The impact of external load resistance on the performance of PAZ3/TPU-TENG (*V*_oc_ and *I*_sc_) was studied under periodic load (10 N), with resistance values ranging from 0.1 MΩ to 1.0 GΩ. The *V*_oc_ increased from 0 to 51 V, while *I*_sc_ decreased from 1.2 to 0 µA. This inverse relationship is due to high internal resistance, which allows high voltage generation but limits current flow. Evaluating the load characteristics of TENG is crucial for ensuring device efficiency in terms of power density. The voltage and current intersect at a specific point where the load resistance is 50 MΩ, as shown in Fig. 10a. This value representing the matched resistance of the device when connecting the PAZ3/TPU device. There were no significant variations in *V*_oc_ and *I*_sc_ were noted while the applied external load resistance was under 0.1 MΩ. The highest power generated by the PAZ3/TPU-TENG was evaluated by substituting the voltage (V) and current (I) values into Eq. (3). The device achieved maximum power of 26 mWm^− 2^ at 50 MΩ. The power density was then determined by dividing the Eq. (4) by electrode active area (A = 0.0004 m^2^). The power density increased with rising input resistance until it plateaued. As per the maximum power transfer theory, highest power is achieved when the external load matches the internal impedance value. The highest power density attained by the device was 0.66 mWm^− 2^ at 50 MΩ, as shown in Fig. 10b. Further increases in the load resistance led to a power density, becoming almost negligible at 1.0 GΩ.

3 $$\:Power\:=V\times\:I$$

4 $$\:Power\:density\:=\:\frac{V\times\:I}{A}$$

### Applications

The real-time applications of PAZ3/TPU-TENG were validated in three distinct ways: as an energy harvester, a low-power electronic operator for miniature devices such as light-emitting diodes (LEDs), and in HCM applications. This demonstrates its ability to capture mechanical energy from the ambient surroundings and convert it into electrical energy to sustainably drive and power up the electronic devices. Additionally, these applications underscore the versatility and potential impact of PAZ3/TPU-based TENG.

### Energy harvesting

The PAZ3/TPU-TENG achieved a power density of 0.66 mWm^− 2^, with a maximum *V*_oc_ and *I*_sc_ of 51 V and 1.2 µA, respectively. This harvested energy was used for two purposes: charging capacitors and powering low-power electronics, such as series of 10 LEDs. However, the voltage produced by the PAZ3/TPU-TENG device was in an alternating waveform, which is incompatible with most portable electronics. To rectify this issue, the alternative voltage produced by the TENG device was changed to direct voltage using a full-wave bridge rectifier. For, functioning as energy harvester for small-scale electronic devices, the PAZ3/TPU-TENG device generated power that could be stored in capacitors. The rectified voltage of 13.6 V, 6.3 V, 4.7 V and 1.5 V were stored in 1.0, 2.2, 4.7 and 10 µF capacitors, respectively over 300 s Fig. 10c. Additionally, the PAZ3/TPU-TENG directly powered a serious connection array of 10 LEDs (shown in supplementary material video S1.) with the operational load set at 10 N (4 Hz), as shown in Fig. 10d. Overall, these demonstrations validate the potential of the PAZ3/TPU-TENG as a promising candidate for powering low-power electronic devices.

### HCM

**Fig. 11:**
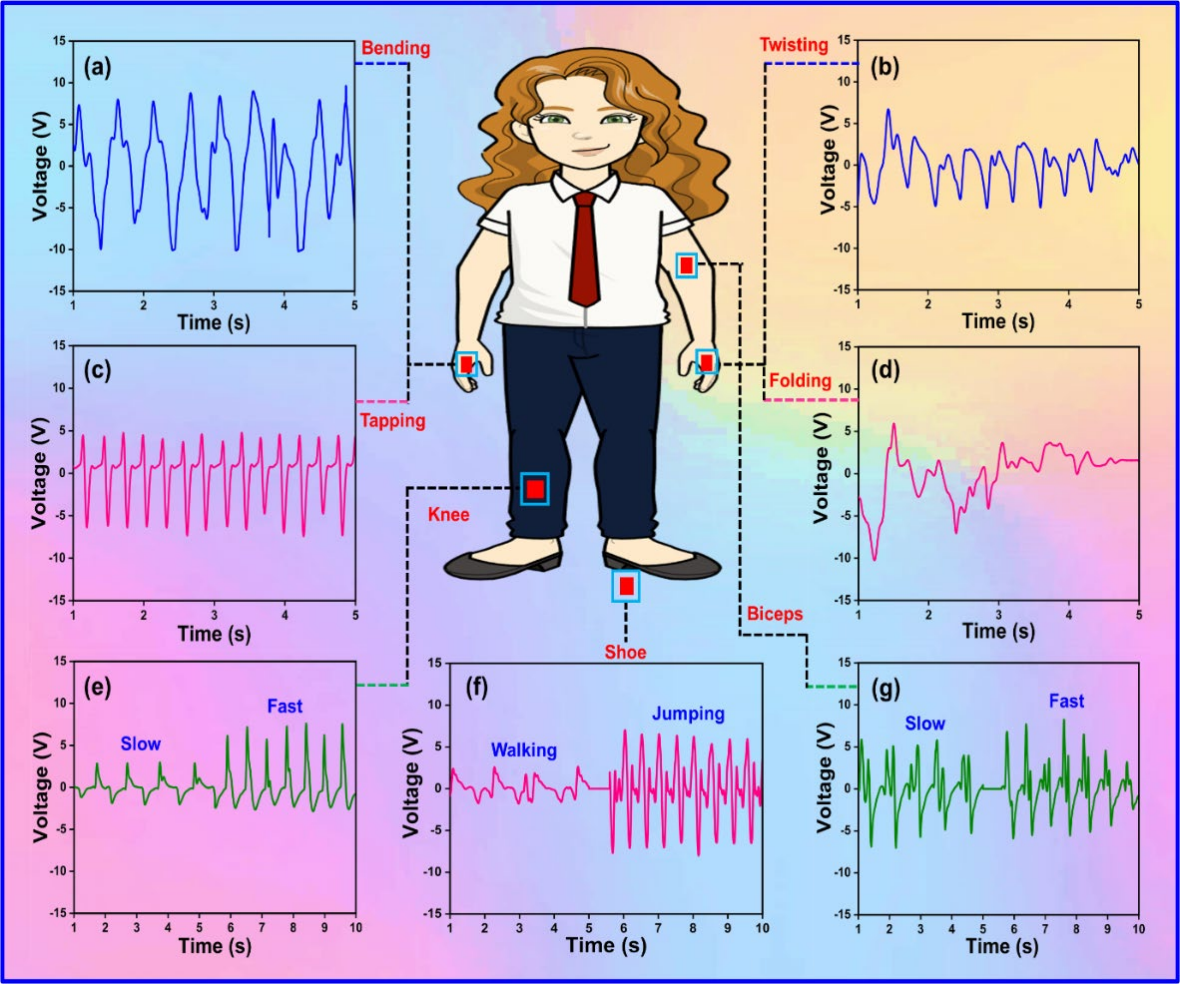
Sensing performance of the PAZ3/TPU-based TENG for various biomechanical movements: (**a**–**d**) Output performance under bending, twisting, tapping, and folding actions, demonstrating the device response to different mechanical deformations, (**e**) Knee movement during slow walking and fast walking, illustrating the device sensitivity to leg motion, (**f**) Shoe insole response during walking and jumping, highlighting the TENG ability to detect foot movements, (**g**) Biceps movements during slow movement and fast movement, showing the device performance in response to arm motion. (Inset: Position of the device placement on various places of the human body for biomechanical sensing).

For HCM applications, the self-powered TENG devices based on PAZ3/TPU can detect human body motion activities. These TENGs were attached to the human body to harvest mechanical energy from human body movements. To evaluate their motion detecting ability, the TENGs, were placed at specific locations, and various motions were performed, including bending, twisting, tapping, folding, knee, shoe sole, and biceps movements. The device generated voltages of 19 V, 11.2 V, 11.4 V, 15.8 V for bending, twisting, tapping, and folding, respectively. The corresponding output is shown in Fig. 11a-d. The improved sensitivity at low-pressure ranges indicates that the TENG device can respond to even the slightest distortions, such as simple finger tapping and movements. Furthermore, variations in walking speed produced different voltage outputs: 4.8 V for low-speed walking and 10.2 V for fast walking when the TENG device was fixed on the knee as shown in Fig. 11e. The device also distinguished between motions when fixed to the leg and shoe insole, as shown in Fig. 11f. It generated, voltage outputs of 3.8 V for walking and 14 V for jumping, likely due to the distribution of the wearer’s body weight across the shoe insole, resulting in a higher response. Figure 11g shown the slow and fast biceps movements, which generated 13 V and 16 V, respectively. Overall, these findings confirm the viability of using the fabricated TENG devices as self-powered devices for human motion monitoring and recognition, indicating its potential abilities for versatile HCM applications.

## Conclusions

In summary, ZnO and Ag-ZnO NPs were synthesized using a chemical co-precipitation method and doped into the PVDF matrix at varying combinations (P0, PZ1, PZ3, PZ5, PAZ1, PAZ3, and PAZ5). The electrospun NFMs were characterized using XRD, FT-IR, and SEM analysis, confirmed the NPs doping in the PVDF matrix. The PAZ3 NFM was optimized to exhibit the highest fraction of *β*-phase and was utilized as TN layer with TPU serving as TP layer for producing a TENG device in CS mode. Triboelectric measurements showed that the PAZ3/TPU-TENG generated a *V*_oc_ of 51 V and *I*_sc_ of 1.2 µA for, which is nearly six times enhanced than P0/TPU (*V*_oc_ of 9.0 V and *I*_sc_ of 0.6 µA). The optimized PAZ3/TPU-TENG demonstrated efficient energy harvesting from abundant and renewable mechanical energy sources. The real-time feasibility of the PAZ3/TPU-TENG was successfully demonstrated by lighting 10 blue color LEDs, energy harvesting, and HCM applications. Therefore, PAZ3/TPU-based TENG underscores its substantial versatility as a promising choice for sustainable energy harvesting and HCM applications.

## Electronic supplementary material

Below is the link to the electronic supplementary material.

Supplementary Material 1

## Data Availability

The data that support the findings of this study are available from the corresponding author upon reasonable request.
