# Supplementary material for: High-performance triboelectric nanogenerators based on Ag-doped ZnO loaded electrospun PVDF nanofiber mats for energy harvesting and healthcare monitoring
Source: Sci Rep. 2025 Jan 27;15:3347. doi: 10.1038/s41598-025-87148-8 (PMC11772763; doi:10.1038/s41598-025-87148-8)
Supplement: Supplementary file 1 — Supplementary Material 1 [file 41598_2025_87148_MOESM1_ESM.docx]

**Appendix A. Supplementary Material**

**High-performance triboelectric nanogenerators based on Ag-doped ZnO loaded electrospun PVDF nanofiber mats for energy harvesting and healthcare monitoring**

**Hema Malini Venkatesan ^a^, Anand Prabu Arun ^a, *^**

^a^ Department of Chemistry, School of Advanced Sciences, Vellore Institute of Technology, Vellore, Tamil Nadu 632014, India

*Corresponding author. E-mail: [anandprabu@vit.ac.in](mailto:anandprabu@vit.ac.in) (A.P.A)


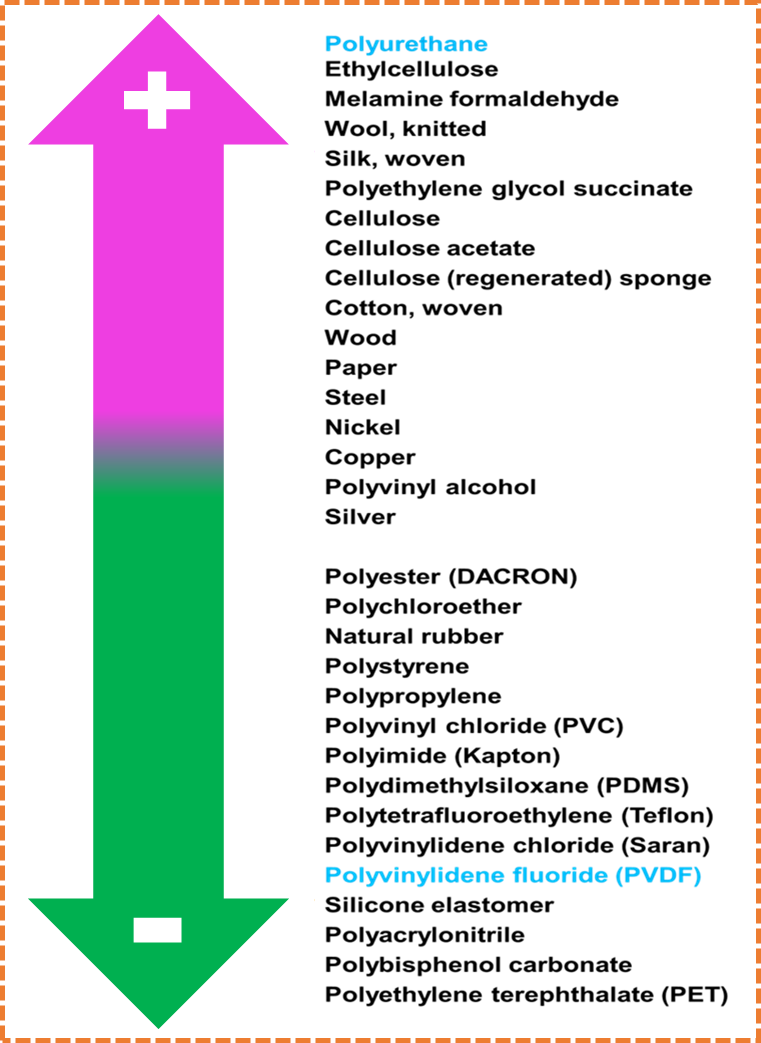


**Figure S1:** Triboelectric material in series follow a tendency to easily lose electrons (becomes positively charged) and gain electrons (becomes negatively charged).


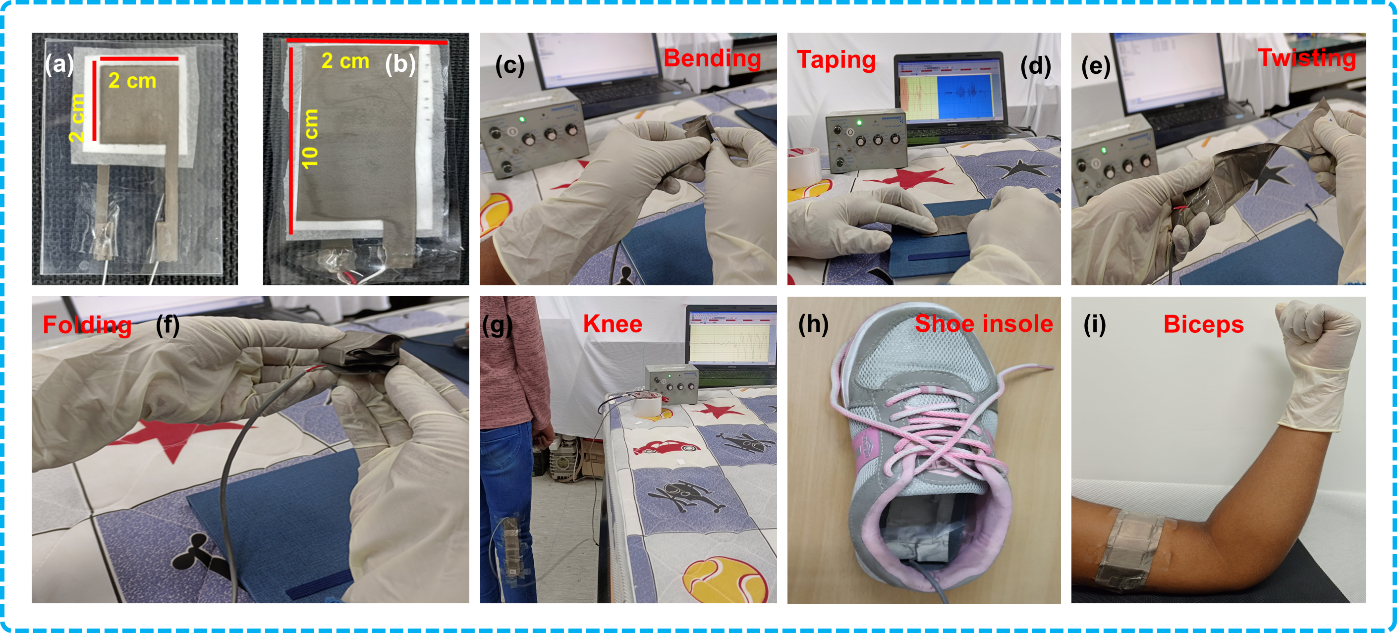


**Figure S2:** (**a and b**) Optical images of the fabricated TENG device in the dimension of 2 x 2 cm^2^ and 10 x 2 cm^2^, respectively, (**c-i**) Various operational process (bending, taping, twisting, folding, knee, shoe insole, and biceps) of the fabricated TENG device for healthcare monitoring applications.


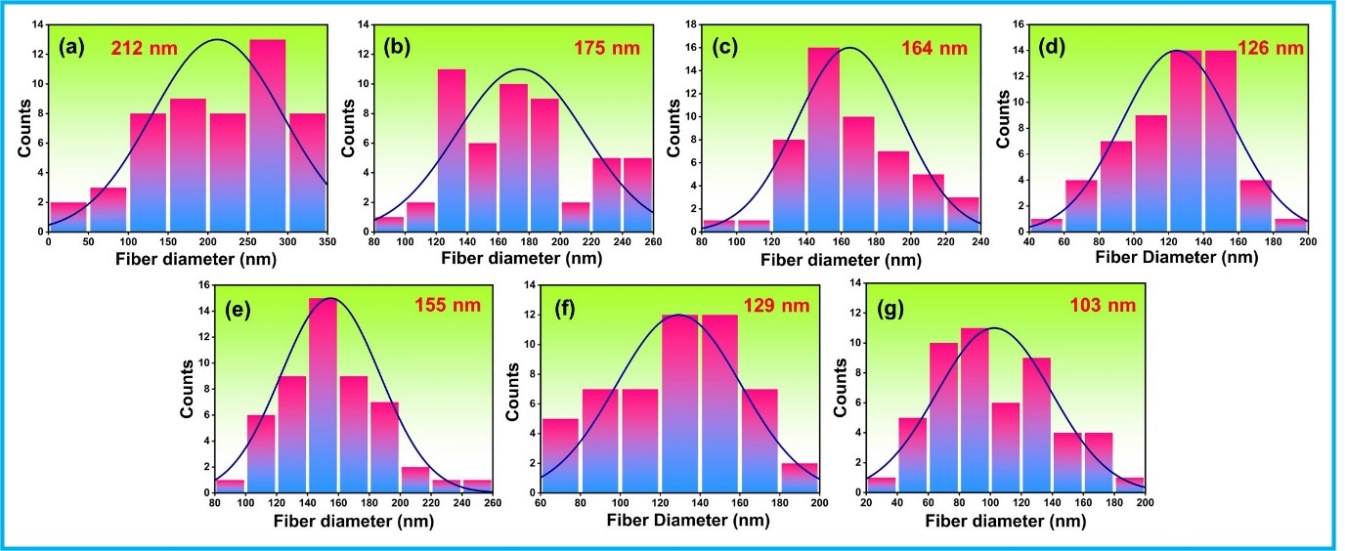


**Figure S3:** (**a-g**) Histogram profile for the average fiber diameter of P0, PZ1, PZ3, PZ5, PAZ1, PAZ3, and PAZ5, respectively.


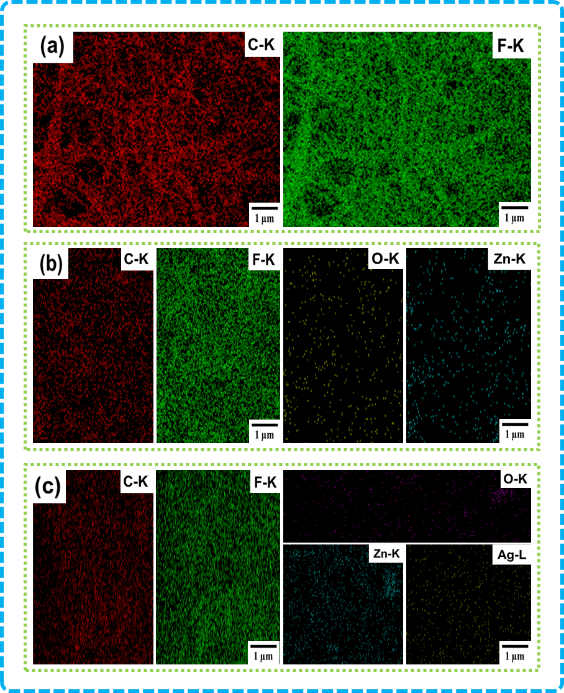


**Figure S4:** EDAX mapping of the fabricated electrospun NFs: (**a-c**) P0, PZ3, and PAZ3 samples, respectively.


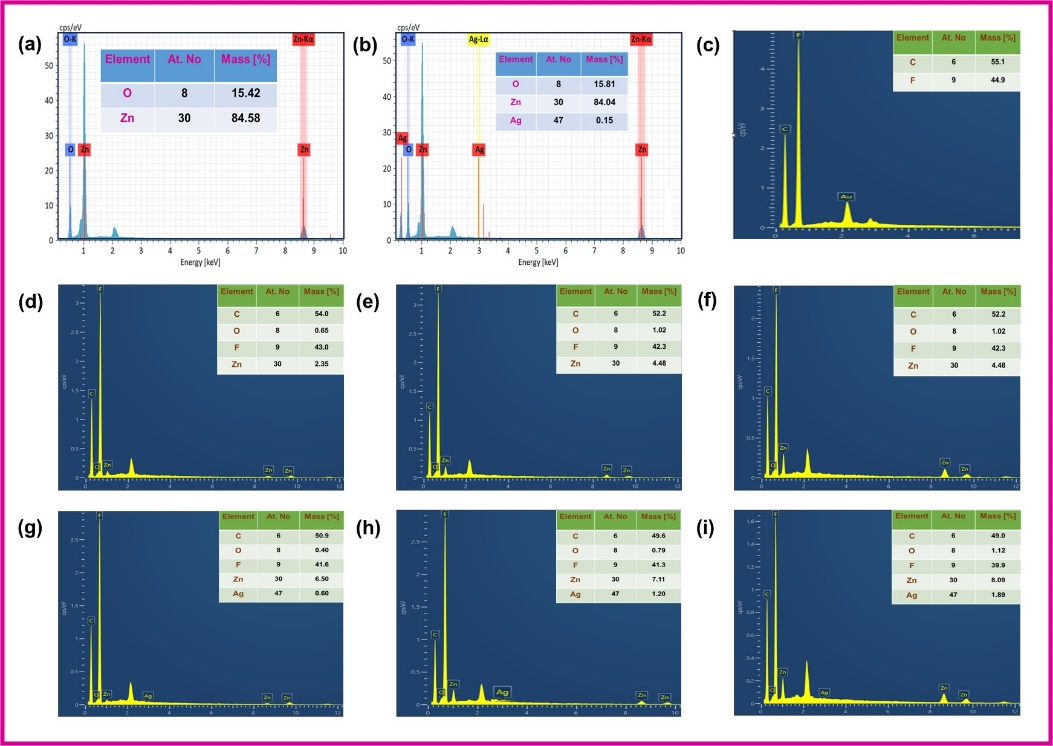


**Figure S5:** (**a and b**) EDAX spectrographs of ZnO, and Ag-ZnO NPs, respectively, (**c-i**) EDAX spectrographs of electrospun NFs: P0, PZ1, PZ3, PZ5, PAZ1, PAZ3, and PAZ5, respectively.


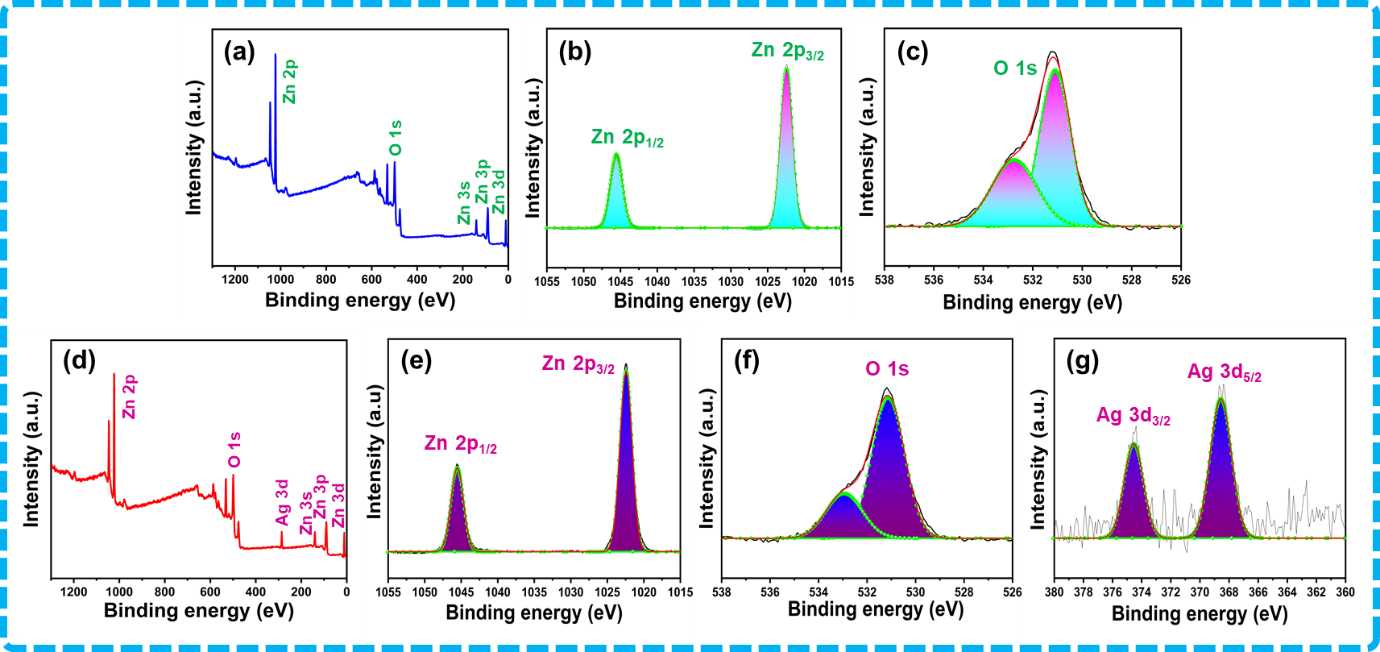


**Figure S6:** (**a**) XPS-survey spectrum for ZnO NPs, (**b**) Zn-2p region, (**c**) Zn-O 1s region, (**d**) XPS survey spectrum for Ag-ZnO NPs, (**e**) Ag-doped Zn-2p region, (**c**) Ag-doped Zn-O 1s region, (**d**) Ag-3d region.


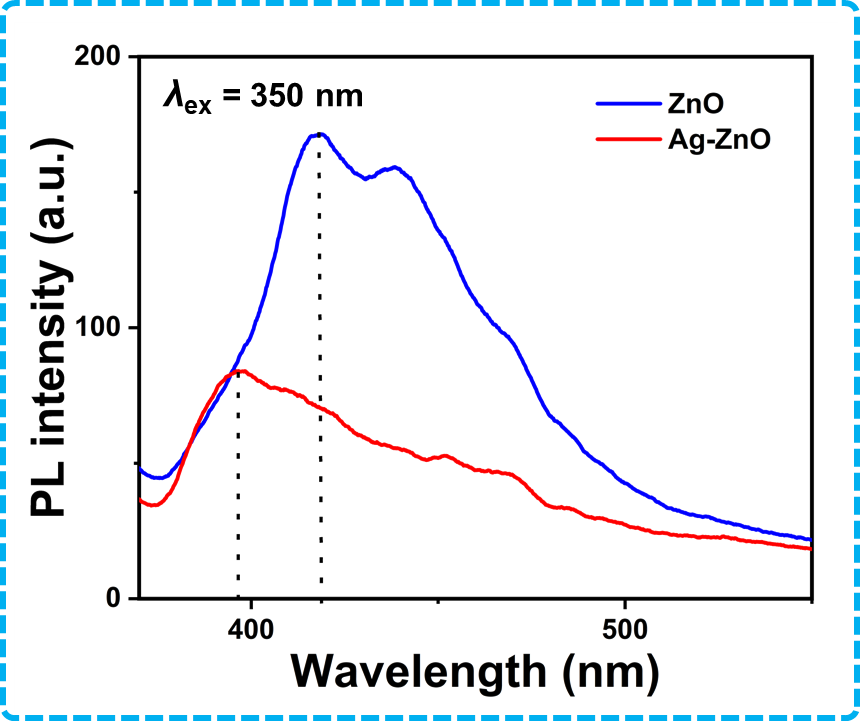


**Figure S7:** Photoluminescence spectra of ZnO and Ag-ZnO NPs.


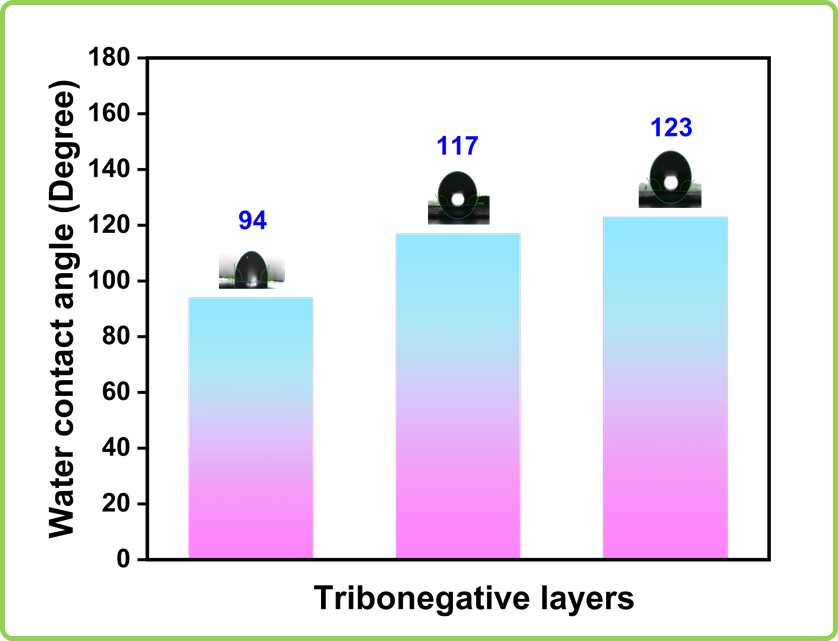


**Fig. S8:** WCAs of P0, PZ3, and PAZ3, respectively.


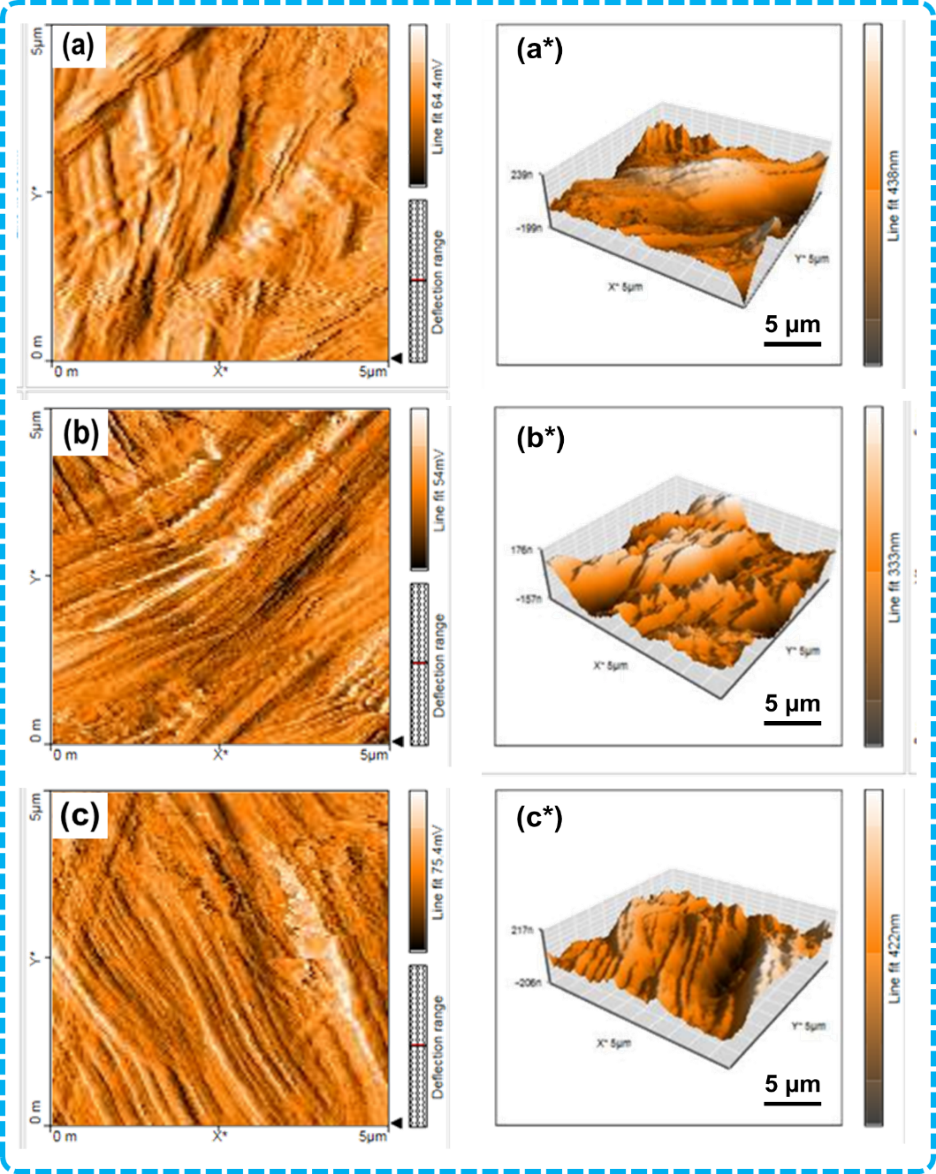


**Figure S9:** (**a-c**) 2-D AFM topographies P0, PZ3, and PAZ3 NF samples, respectively, (**a*-c***) 3-D AFM topographies P0, PZ3, and PAZ3 NF samples, respectively.


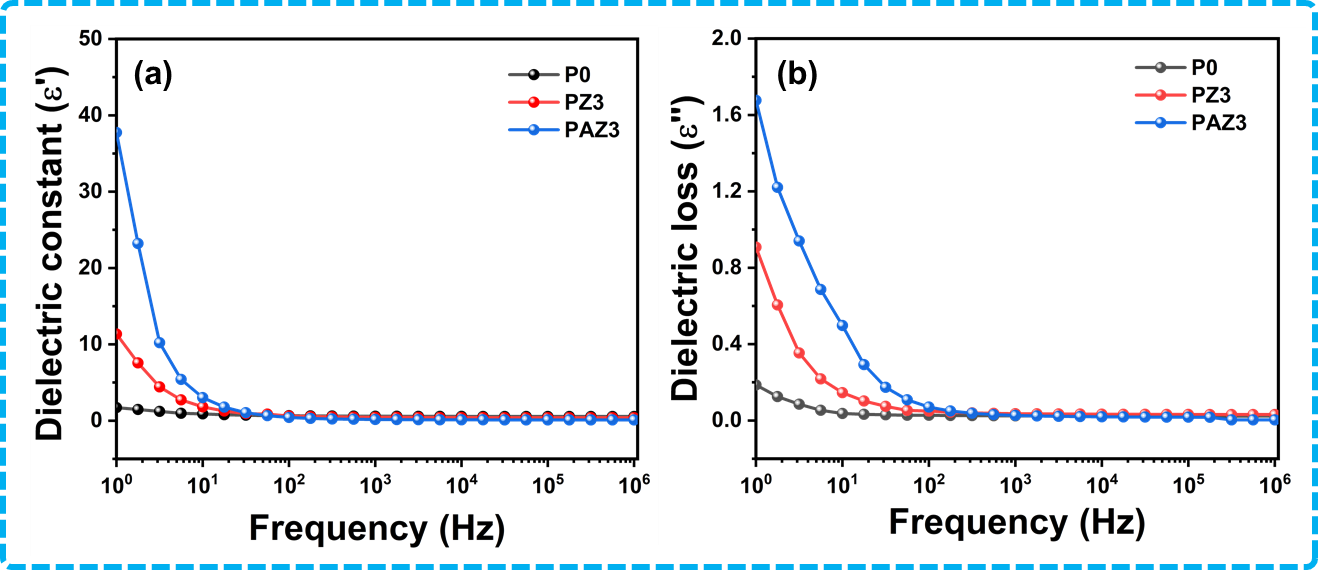


**Figure S10:** (**a**) The dielectric constant for P0, PZ3, and PAZ3 NF samples, (**b**) The dielectric loss for P0, PZ3, and PAZ3 NF samples.


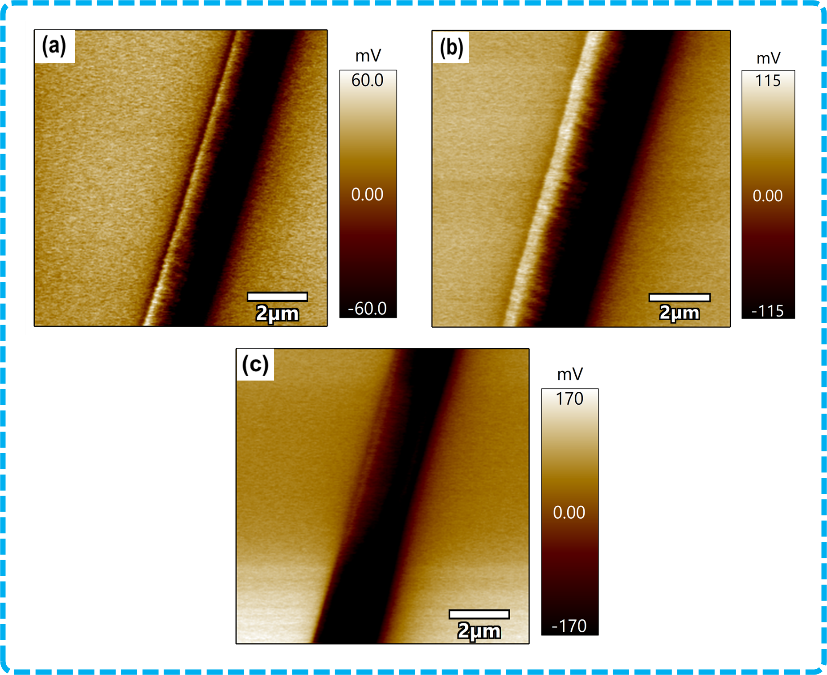


**Figure S11:** (**a-c**) SKPM surface potential profiles of P0, PZ3, and PAZ3, respectively.


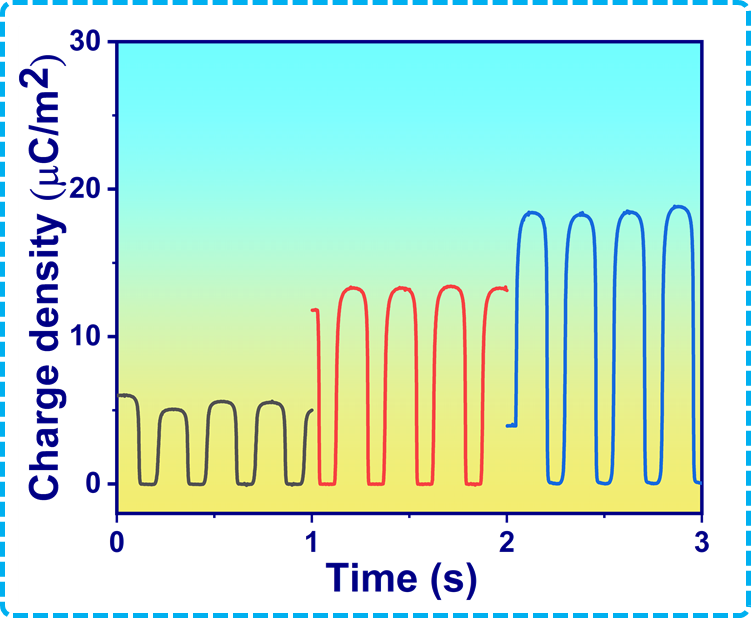


**Figure S12:** Surface charge density of P0, PZ3, and PAZ3 NF samples.

| **S. No** | **TN layer** | **TP layer** | **Output Voltage (V)** | **Ref.** |
| --- | --- | --- | --- | --- |
| 1. | PVDF | TPU | 3.7 | ^1^ |
| 2. | PVDF/Ar.HBP-2 | TPU | 6.27 | ^2^ |
| 3. | PVDF/CuO | TPU | 7.5 | ^3^ |
| 4. | PVDF/CF | TPU | 8.0 | ^4^ |
| 5. | PVDF-Si.HBP-G3-10 | TPU | 39.7 | ^5^ |
| 6. | PVDF/Ag-ZnO | TPU | 51 | Present Work |

**Table S1:** Comparison table of various studies used PVDF and TPU as the triboelectric pair

.

**Table S2:** FTIR: Fraction of *β*-phase for the prepared samples.

| **S. No.** | **Sample Code** | **PVDF: NPs** | **Absorption Intensity** | | **F(*β*)%** |
| --- | --- | --- | --- | --- | --- |
|  |  |  | **A*_α_*** | **A*_β_*** |  |
| 1. | P0 | 10:0 | 0.0112 | 0.0249 | 64 |
| 2. | PZ1 | 10:1 | 0.0833 | 0.4031 | 79 |
| 3. | PZ3 | 10:3 | 0.0912 | 0.5064 | 82 |
| 4. | PZ5 | 10:5 | 0.0723 | 0.2910 | 76 |
| 5. | PAZ1 | 10:1 | 0.0420 | 0.2658 | 83 |
| 6. | PAZ3 | 10:3 | 0.0320 | 0.2973 | 88 |
| 7. | PAZ5 | 10:5 | 0.1920 | 1.0437 | 81 |

**Table S3:** AFM topographical parameters of the TN layers.

| **Samples** | **S*_a_* (nm)** | **S*_q_* (nm)** | **S*_y_* (nm)** | **S*_q_*-S*_a_* (nm)** |
| --- | --- | --- | --- | --- |
| P0 | 47 | 56 | 351 | 9 |
| PZ3 | 53 | 75 | 448 | 22 |
| PAZ3 | 62 | 89 | 625 | 27 |

*S*_a_ (surface roughness), *S*_q_ (root mean square surface roughness) and *S*_y_ (valley depth) values.

**References**

1. Le, B. *et al.* Flexible piezoelectric PVDF/TPU nanofibrous membranes produced by solution blow spinning. *J. Mater. Res. Technol.* **24**, 5032–5041 (2023).

2. Sathiyanathan, P. *et al.* Piezoelectric-piezocapacitive hybrid sensor based on electrospun Poly(vinylidene fluoride)-Poly(octafluoropentyl acrylate)-sulphonated Poly(phenylene sulfide) blend nanofiber. *Sensors Actuators, A Phys.* **331**, 112993 (2021).

3. Amrutha, B. *et al.* Fabrication of CuO-NP-doped PVDF composites based electrospun triboelectric nanogenerators for wearable and biomedical applications. *Polymers (Basel).* **15**, 2442 (2023).

4. Venkatesan, H. M. *et al.* Cobalt ferrite-embedded polyvinylidene fluoride electrospun nanocomposites as flexible triboelectric sensors for healthcare and polysomnographic monitoring applications. *Nano Energy* **129**, 110003 (2024).

5. Indumathy, B. & Anand Prabu, A. Electrospun PVDF/Si-HBP of 3^rd^ gen based nanoweb as flexible and self-powered TENG for real-time energy harvesting. *Mater. Sci. Eng. B* **302**, 117216 (2024).
